# A universal hydro-mechanical coupled behavior model for clay-bearing strata—Molecular-level simulation approach

**DOI:** 10.1038/s41598-023-47402-3

**Published:** 2023-11-14

**Authors:** Muhammad Abdul Waheed, Omar S. Baghabra Al-Amoudi, Mohammed A. Al-Osta, Habib Ur-Rehman Ahmed

**Affiliations:** 1 Department of Civil and Environmental Engineering, King Fahd University of Petroleum and Minerals (KFUPM), 31261 Dhahran, Saudi Arabia; 2 Interdisciplinary Research Center for Construction and Building Materials, KFUPM, 31261 Dhahran, Saudi Arabia

**Keywords:** Nanoscale materials, Hydrogeology, Geomorphology

## Abstract

Clay minerals in soils and rocks exhibit large volume change upon interaction with water and this behavior becomes even more complex when the strata are being stressed by the engineering and environmental loads. Therefore, a realistic prediction of the hydro-mechanical behavior of the clay-bearing strata is always a challenge due to their coupled swelling-mechanical response in the cases of geotechnical and geoenvironmental engineering problems, nuclear waste storage in clay-bearing rock repositories, shale gas extraction, and other uses of clay in the manufacturing industry. All the existing behavior models have restricted applications in the engineering and other fields of practice mainly due to the partial consideration of the structure and fabric of clay-bearing strata in the model formulation. In this study, a hydro-mechanical behavior model has been formulated using the parameters acquired from the molecular-level simulations and modeling of the volume change and stress–strain behavior of the clay-bearing structure. The Molecular Mechanics and Molecular Dynamic simulations were performed on the natural structure of the clay-bearing strata formulated using Monte Carlo technique. The mathematical model, developed from the simulation results, can predict the overall hydro-mechanical behavior of clay-bearing strata for all possible combinations of clay minerals, non-clay minerals, salts causing cementation of the soil/rock structure, confining pressures, and the induced strain levels. The developed model has successfully been validated through laboratory and field testing on the clay-bearing strata in both the elastic and elasto-plastic regions of the stress–strain behavior and also from the data of two (02) swelling clays (MX-80 and FEBEX Bentonite) from the existing literature, supporting the universal nature of the developed behavior model.

## Introduction

Clay-bearing strata are characterized by the presence of the swelling clay minerals in the soil or rock structures. Clay minerals present in soils and rocks exhibit a high degree of volume change upon interaction with water and this behavior becomes even more complex when the strata are being stressed by the engineering and environmental loads. Clay-bearing strata are known to be problematic because of their volume change characteristics resulting from a continuous variation in the moisture regime of the unsaturated and partially saturated zones. These volume changes are accompanied by variations in the mechanical behavior of clay-bearing strata. The mechanical properties of natural and compacted clays are critical in the foundations' design and containment barriers for nuclear waste^1–5^. Foundations of the buildings are generally placed in partially saturated soil layers of the vadose zone. The increase in the moisture levels under the building foundations due to reduced evaporation, percolation of surface water(s), and the possibility for leakage from utilities will lead to significant changes in the mechanical properties of these soils. The hydro-mechanical behavior of compacted clays is also critical in the design of clay barriers for nuclear waste. The clay barrier is initially placed in an unsaturated state and is exposed to moisture fluctuations during its lifetime caused by heat-emitting waste and hydration from the parent/host rock. Similarly, slopes and excavation work in mudrocks/clayey rocks also require special attention to study the hydro-mechanical behavior^6^. Excavations in clayey rocks create unloading conditions (reduction in the confining stress) and expose the surrounding material to atmospheric interaction causing cyclic changes in the moisture levels. These changes in environmental and loading conditions lead to swelling of the exposed clayey rocks and the corresponding reduction in stiffness and strength. Further, the clays have applications in agriculture soils, landfill liners, and even in the field of medicine^7^. Therefore, understanding the hydro-mechanical behavior of clay-bearing strata is important for the application of clay-bearing soils/rocks in various disciplines and civil engineering.

The structure of the natural and compacted clay-bearing strata is very complex since they constitute different minerals with random arrangements and complex interactions of the particles ranging from millimeters to nanometers^8,9^. The overall material behavior is controlled by the structure and fabric of clay-bearing strata created by several factors such as clay-water-ion interactions, relative distribution and proportion of clay and non-clay particles, density, and moisture conditions^10^. Due to above-reported factors, the realistic modeling of clay-bearing soils/rocks has been very challenging for geotechnical engineers. Therefore, clay minerals are nano-materials and molecular-level simulations approach can be applied to determine the overall performance of clay-bearing soils/rocks.

Several macro-level reports^2,6,8,11–23^, as well as molecular-level studies^7,24–34^, have been conducted to model the volume change and mechanical performance of clay-bearing strata. All macro-level constitutive models are applicable to specific behavior(s) and cannot be generalized in the form of one universal model. The macro-level models also do not explicitly incorporate realistic nano-level clay interactions with water and ions in their formulations. On the other hand, the existing molecular-level models for clay-bearing soils/rocks have mainly used a single clay crystallite in their simulations without incorporating the natural fabric and structure of clays. In addition, the existing molecular-level models also lack in formulating a mathematical model to assess the volume change and mechanical behavior. In the light of the above-described limitations of macro and molecular level models, it is imperative to perform realistic modeling of clay-bearing strata at the molecular level to predict the hydro-mechanical behavior with due consideration of the clay interactions with water and ions at the nano-level and linking it to the macroscopic level by considering all possible variations in the structure and fabric of clay-bearing soils/rocks.

In the current report, the authors present a comprehensive molecular-level simulations-based hydro-mechanical model for clay-bearing strata, which could be utilized for all possible combinations of clay and non-clay minerals with cementing agents. The model developed in this study is based on the basic intrinsic parameters utilized in various types and kinds of clay-bearing soils/rocks available worldwide. This research is comprised of three major levels: molecular-level simulations; model formulation; and model validation. Molecular-level simulations were carried out using material studio software followed by the model formulation using simulation results. Validation of the model was then performed through field and laboratory testing in the current study and utilizing the available database from the literature. All these phases of the study are discussed in the subsequent sections.

## Molecular-level simulations

### Model construction

The natural composition of clay-bearing strata is very complex owing to the presence of swelling clay minerals such as Na-montmorillonite (Na-Mt), non-swelling clay minerals such as kaolinite and interaction of these clay minerals with other non-clay minerals like quartz, gypsum, calcite and other salts. Cations and anions from these salts interact with the swelling clay minerals and cause cementation. Quartz acts as filler in the pore spaces and do not contribute to the volume change process. Furthermore, macro, micro and nano level pores exist in the clay-bearing strata with water and air phases. The overall volume change and mechanical behavior of the clay-bearing strata are thus controlled by the swelling clay minerals, density, degree of saturation and cementation between clay minerals. All such possible clay-water-ion compositions and interactions in the clay-bearing strata are simulated in this study through Materials Studio software^35^ using molecular dynamics (MD), molecular mechanics (MM), and Monte Carlo (MC) techniques.

**Figure 1:**
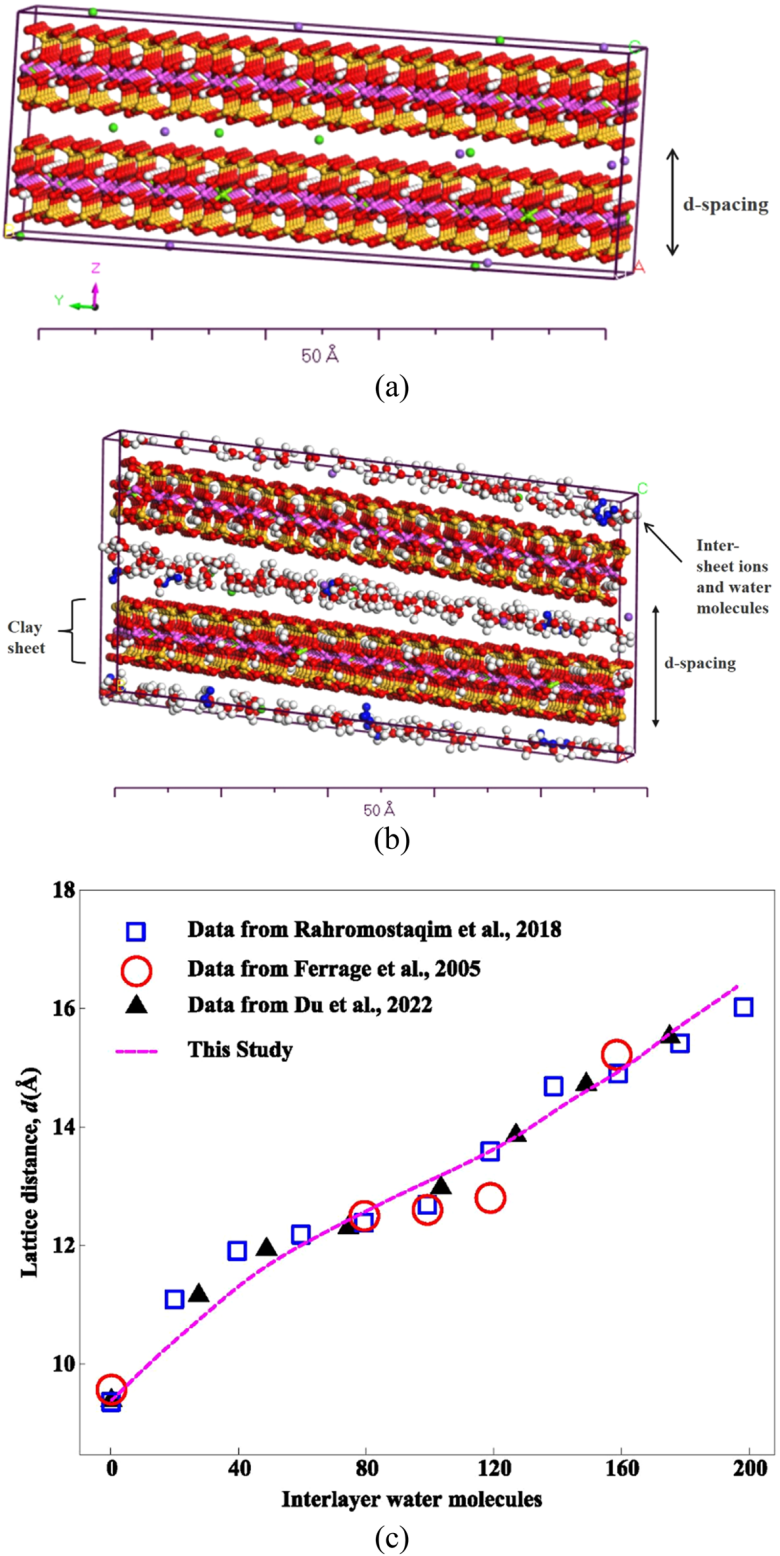
(**a**) Single crystallite of Na-Montmorillonite, (**b**) Sorption of water on single crystallite of Na-Mt, (**c**) Increase in lattice distance of Na-Mt after water sorption.

It is well established that the swelling mechanism of clays is governed by the interactions and processes among the nano-level clay particles, called as crystallites^31,36–39^. The swelling response, in turn, defines the mechanical performance of these soils/rocks^7,27–29,32^. In this study, the swelling-mechanical response of clay-bearing strata is modeled through molecular-level simulations of Na-montmorillonite with different cation exchange capacities (54, 90, and 144 meq/100 g). Na-montmorillonite has a tetrahedral-octahedral-tetrahedral (TOT) structure in which silica tetrahedral sheets are composed of one silicon atom surrounded by four oxygen atoms and the octahedral sheets contain aluminum or magnesium that are octahedrally connected by hydroxyls and oxygen atoms. The clay particles form by stacking of these sheets with dimensions of an order of nanometer and a length or width to thickness ratio of about 2000:1^9^. Based on the already published values in literature^40–45^ and XRD findings by Ahmed and Abduljauwad^7^, a basic crystallite size of 2·6 × 5·4 × 2·0 nm was selected for the simulation of Na-montmorillonite (Fig. 1a). These individual crystallites were then grouped in a periodic unit cell, utilizing the MC technique to form bigger clay particles. The clay particles then combine to form aggregates that join with other non-clay particles to generate the overall fabric and structure of clay-bearing soils/rocks having micropores between clay particles, inter-aggregate and intra-aggregate pore spaces. Most of the unit molecules used in this study were obtained from the Nanoscale Simulation Laboratory at the University of Akron, USA^46^. These unit molecules consist of Na-montmorillonite with different cation exchange capacities (54, 90, and 144 meq/100 g), water, Calcite, Gypsum, and Pyrophyllite. Some other molecules were also drafted using Materials Studio software. All these molecules' charges were validated using the method of charge equilibration QEq^35^.

### Simulation parameters

One of the basic steps in molecular-level simulations is the sorption of water molecules in the clay crystallites to simulate the swelling process. The sorption module of the software was used for the sorption of water on the selected clay crystallites using MC method (Fig. 1b). The MC method checks the fixing of the defined number of water molecules at best-fit places. The MC uses the random placement principle to fit water molecules in the best possible configuration through 'exchange', 'conformer', 'rotate,' 'translate,' and 'regrow' ratios. Various values of these parameters were tried and the resulting clay-water configurations were verified against the known behaviors like hydration radii of Na^+^ cations and the expansion of clay lattice with change in water content in the interlayer. The finally selected values of these parameters, verifying these known behaviors, were 0·39, 0·20, 0·20, 0·20, and 0·20, respectively, against probabilities of 0·39, 0·20, 0·20, 0·20, and 0·20. The modified universal force field was used in the simulations through the Forcite module of the software. Berendsen thermostat, Berendsen barostat, and NPT (constant number of particles, pressure, and temperature) ensemble were selected to perform the simulations. A decay constant of 0.1 ps was used in both the Berendsen thermostat and barostat, with a temperature of 298 K in the thermostat and a pressure of 100 kPa in the barostat. After the water molecules' sorption, the system's energy was minimized using geometry optimization and the MD technique in the Forcite module^35^.

As explained earlier, the verification and calibration of above-described parameters in the simulation study was carried out using the relationship between the moisture content and d-spacing in Na-montmorillonite. It has been found through previous studies^31,33,36^ that d-spacing increases with an increase in water molecules in the clay layers. The increase in d-spacing of the study model with water molecules is shown in Fig. 1c and is found to be in good agreement with previous experimental, numerical, and simulation studies^31,33,36^.

### Simulation methodology

A six (06) step simulation methodology comprising: (i) water molecules sorption on the individual clay crystallites, (ii) salts sorption, such as calcite and gypsum on clay crystallites, (iii) creation of soil fabric in the loose state by random sorption of four crystallites in the unit periodic cell, (iv) compaction of the soil to the required density, (v) stress relief of the compacted unit cell, and (vi) sorption of water to produce different swelling stages, was adopted using selected variations of cation exchange capacity (CEC), exchangeable cations, density, moisture content, and salts. These steps of the simulation program were repeated for three CEC changes in Na-montmorillonite (144 meq/100 g, and CECs of 90, 54) and subsequent variations in density, moisture condition, exchangeable cation, and salt. In the simulation for Na-montmorillonite at three different CECs (Na as 100% exchangeable cation), the clay particles were compacted at 0.01, 0.1, and 1.0 GPa at moisture contents of 10, 20, 30, and 40%. Cementation agents, such as Calcite (CaCO_3_) and Gypsum (CaSO_4_.2H_2_O), were added to Na-montmorillonite at 10 to 20%. Simulation permutations with these salts were performed at 10% for Calcite and 10 and 30% for Gypsum. Exchangeable cations (i.e., Na^+^, K^+^, Mg^+2^, and Ca^+2^) were also varied at 20, 40, and 60% with different combinations to investigate their effect on swelling and subsequent mechanical behavior.

**Figure 2:**
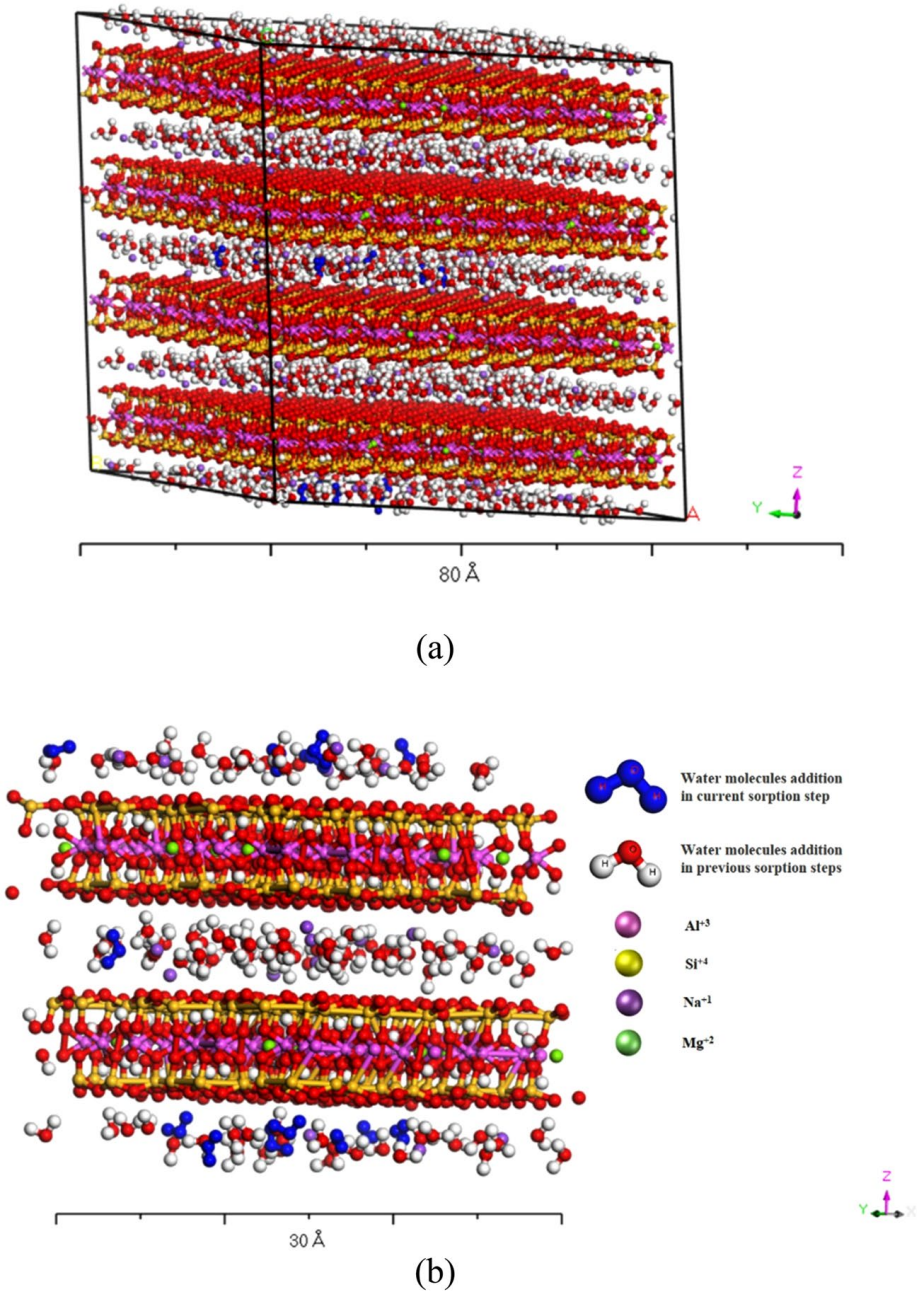
(**a**) Water sorbed particles of Na-montmorillonite, (**b**) A close up view of Na-Montmorillonite particle after water and cations sorption.

The sorption of water molecules on the selected clay crystallites was achieved using the sorption module. After sorption of a maximum number of possible water molecules in every 25,000 steps, the system's energy was minimized using the software's molecular dynamics technique in the Forcite module. After the sorption of water molecules on single clay crystallites, the individual ions, e.g., Ca^+2^, SO_4_^–2^, etc., were sorbed on the water-bearing clay crystallites to simulate the process of cementation due to salts. A typical post-sorption particle of Na-montmorillonite is shown in Fig. 2a, and an enlarged view showing various inter-layer and intra-layer cations is provided in Fig. 2b.

**Figure 3:**
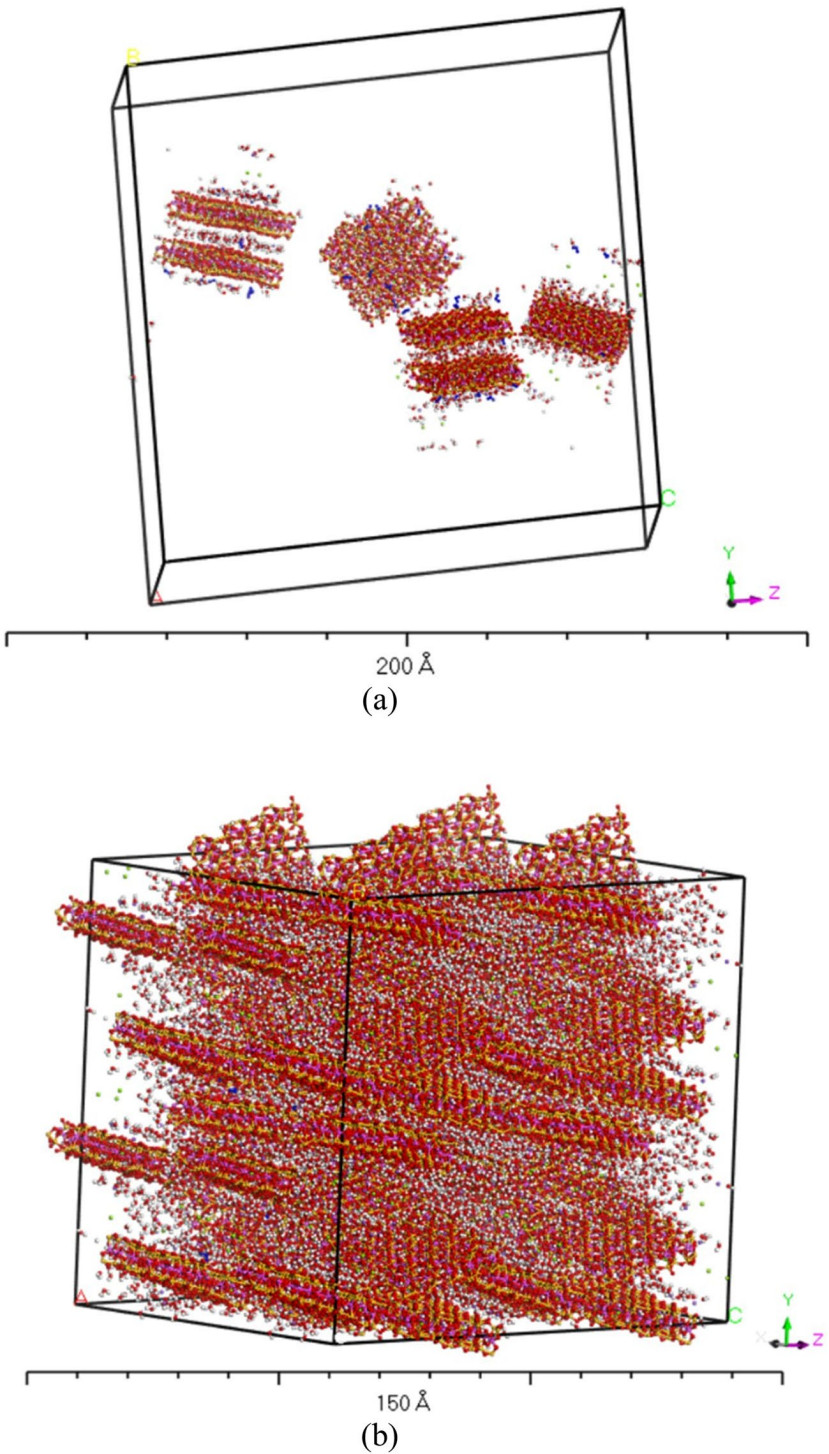
(**a**) Cubic unit periodic cell representation of four water sorbed crystallites, (**b**) Dispersed fabric at 0.001 GPa pressure and 10% moisture content.

During the deposition process, the clay particles first pack in a loose state and then get compacted under the consolidation process. Therefore, it was essential to simulate the clays' fabric at both stages. Using the Sorption module, simulation of the loose state is performed through random sorption of four crystallites with periodic boundary conditions in a 12·5 × 12·5 × 12·5 nm cubic unit cell (Fig. 3a). These loose particles were then compacted to the maximum achievable density using the NPT ensemble in the Forcite molecular dynamics module (Fig. 3b). Confining pressures of 0.01, 0.10, and 1.0 GPa were employed to model the different types and levels of laboratory compaction pressures and geological pressures. Each simulation was run for a varying period (ranging from 10 to 30 ns). The simulation time period was extended to a maximum limit where it was made sure that no more increase in density, under that confining pressure, is achievable and it has become constant. This is the stage where the particles have achieved the maximum possible packing by occupying all the possible empty spaces. This packing is governed by the size, shape and gradation of the clay particles.

Clay-bearing strata are subjected to the release of geological pressures (stress relief), making them overconsolidated in nature and prone to swelling. In the previous step, the Forcite MD module was run at a confining pressure of 0·001 GPa to simulate the stress relief of the compacted unit cell. The selected pressure of 0·001 GPa could be considered a representative of the over-consolidation pressure in most of the clay-bearing soils/rocks. The clay particles mixture after stress relief was subjected to sorption of water molecules to create different swelling stages. The process was repeated for different percentages of moisture content.

In this study, each absorption/adsorption run was carried out using Monte Carlo technique. In this simulation, the water molecules start absorbing/adsorbing to the highest energy points in the clay structure and continue till all the energy (water deficiency) points are covered by water molecules. Hence, in this manner, the simulation is continued till the adsorption/absorption process of the water molecules is completed.

### Simulation outcomes

The mechanical behavior model formulated in this study considered cohesive energy density (CED), density, and moisture content as three (03) state parameters. The Forcite module of Material Studio software was used to determine CED and moduli values for all likely variations in the soil/rock structure.

CED is the energy required to separate molecules from each other and is given as:

1 $$ {\text{E}}_{{{\text{coh}}}} = \, - {\text{E}}_{{{\text{inter}}}} = {\text{E}}_{{{\text{intra}}}} - {\text{ E}}_{{{\text{total}}}} $$

where E_coh_ is the cohesive energy of all molecules in Joules, E_total_ is the total energy of the system, and E_inter_ and E_intra_ are the intermolecular and intramolecular energies, respectively in Joules. The cohesive energy per unit volume defines the CED and is generally represented in units of J/cm^3^. It is the measure of mutual attraction between the molecules^7^.

Both CED concept and volume change processes of clay-bearing strata are quite analogous to each other and, therefore, CED parameter is found to correlate well with all possible variations in the structure of clay-bearing soils/rocks resulting from changes in density, moisture content, CEC, and exchangeable cations^7^.

The CED value depends on the net result of all the interactions taking place in a clay-water-ion system including ionic hydration, adsorption, double layer repulsion, electrostatic attractions and van der Waals interactions^7^. The CED parameter varies with the changes in density, moisture content, cementation, exchangeable cations, and CEC and is directly/indirectly associated with all of these variables. In this study, the clay crystallites with high CEC have been observed to yield high CED values as compared to low CEC crystallites for the same density and moisture. This is because of the more number of hydrogen bonds in high CEC clays due to the higher charge deficiency centers which results in more electrostatic attractions in clay particles. The CED also shows an increasing trend with the increased bivalent cations and cementation. This is due to the extra bonding created by the cations and anions of cementing agents such as Calcite, Gypsum, etc., and/or exchangeable cations other than sodium. The clay crystallites with high densities have been found to achieve higher CED values due to close proximity of the crystallites or lesser moisture contents. The increase in CED owes to the additional attractions due to close range attractive forces on the crystallites. However, as the clay particles come closer due to the high electrostatic attraction, the van der Waals forces become repulsive resulting in additional expansion. For the same CEC, the higher water content results in a decrease in the CED value due to balancing of charge deficiency centers by water molecules thereby lowering the electrostatic attractions. In conclusion, the CED parameter depends on density, moisture content, cementation, exchangeable cations, and CEC and controls the swelling and mechanical response of clay-bearing strata.

**Figure 4:**
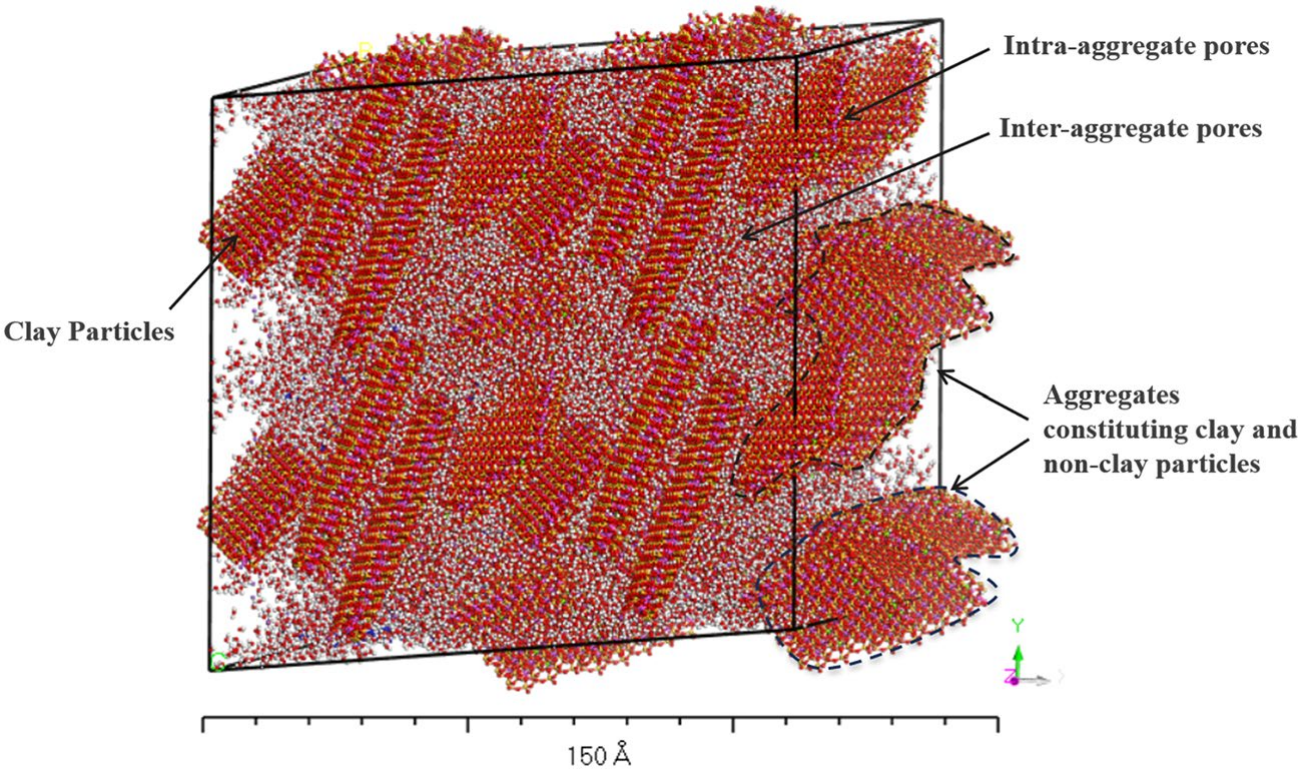
Three-dimensional view of multiple unit cells after CED and modulus simulations of Na-montmorillonite with non-clay particles (water content = 10%, strain level = 1.0%).

The moduli values were determined using the Forcite module in the material studio at each swelling stage. The constant strain method was used for the modulus determination at strain levels varying from 0.001% to 10% in four steps against each strain level. The software uses the Voigt-Reuss-Hill (VRH) approximation to determine the moduli values. The adopted simulation methodology in this study considered all possible variations in the strain levels (0.001% to 10%) that are generally observed in geotechnical engineering and, therefore, it is applicable to assess moduli values in the elastic and elasto-plastic regions of stress–strain behavior of clay-bearing strata. A three-dimensional view showing the clay-bearing strata structure with inter-aggregate and intra-aggregate pore spaces after CED and modulus simulations is shown in Fig. 4.

There could be directional arrangement of the clay particles during the compaction process. 3D views of the repeated cells shown in Figs. 3b and 4 reveal various nature of fabrics consisting of various directional arrangement of clay particles. The mechanical properties determined for these 3D fabrics are representative of the three-dimensional stress case and hence covers the effects of the anisotropy if there is any.

## Nano-level hydro-mechanical model

As explained earlier, CED can be effectively used in predicting the volume change and mechanical behavior of clay-bearing strata due to its sensitivity to changes in CEC, exchangeable cations, cementation, density conditions, and water. Therefore, this study has used it as a state parameter to predict the swelling behavior and mechanical properties of clay-bearing strata at different stages of swelling.

**Figure 5:**
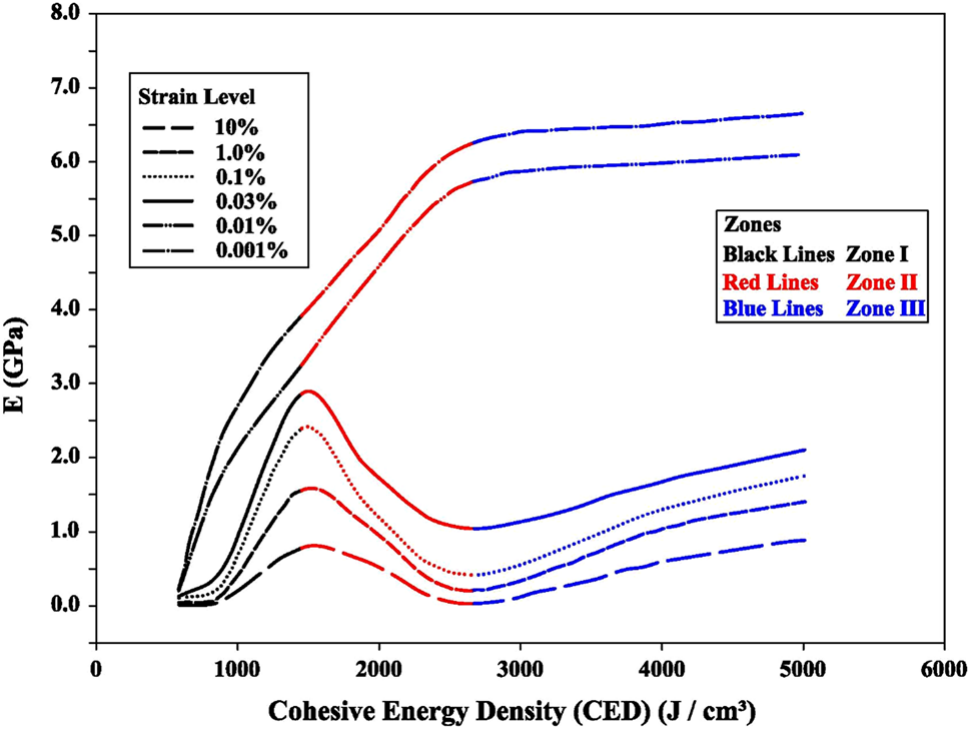
Mechanical behavior model plot for swelling soils.

The Forcite module of Material Studio software was used to determine the CED and the corresponding modulus values. The simulation permutations were repeated for the adopted cases with varying density, cementation, moisture conditions, and exchangeable cations at strain levels of 0.001% to 10%. The outcomes of the simulations are plotted as a mechanical behavior model in Fig. 5.

The mechanical behavior model has been developed using a molecular level simulations approach, but the resulting structure and fabric of clay-bearing strata modeled in the software consists of both nano and micro-level pores, which are typically present in any overconsolidated hard clay/clayey rock structure. This is evident from the three-dimensional (3D) structure created using periodic boundary conditions (Fig. 4). Periodic boundary conditions create multiple unit cells of the resulting structure by replicating the model in all three dimensions infinitely. The amount of large-sized pores is almost minimal in the case of heavily overconsolidated clay-bearing soils/rocks because of the high densities, while there could be relatively high percentages of macropores in low density strata.

Two (02) types of trends can be observed from the mechanical behavior model plot (Fig. 5), one at small strain levels (0.01% and 0.001%) and the other at large strain levels (0.03% to 10%). Small strains range is applicable to foundations subjected to dynamic loads and corresponding field geophysical tests (i.e., in-situ cross-hole and downhole tests etc.). The large strain range is related to foundations, retaining walls, and conventional soil testing approaches^47,48^.

Three zones are obvious at a large strain level (Fig. 5). Zone I is bordered by CED values of 584 J/cm^3^ and 1450 J/cm^3^. In this zone, the modulus values demonstrate an increasing trend with the raise in CED. This is mainly attributed to the increase in CEC with no contribution from the cementation between the particles. There is a tendency of reduction in the modulus values with an increase in CED in Zone II, bounded by CED values of 1450 J/cm^3^ and 2640 J/cm^3^. Theoretically, the increase in CED in Zone II is only possible due to either the reduction in moisture content or an increase in cementation due to presence of salts such as Calcite, Gypsum, etc. It has been observed in molecular simulations that the presence of cementation in clay-bearing strata yields higher CED values than the limiting value of 2640 J/cm^3^ for Zone II. Therefore, it can be interpreted that the increase in CED in Zone II is primarily due to a reduction in moisture content (dry nature of soil). The reduction in moisture content will cause the crumbling of dry clays and, ultimately, loss of strength and stiffness. In Zone III (CED > 2640 J/cm^3^), there is again a trend in the rise of the modulus values with the increase in CED. The increase in CED in this zone is caused by an increase in cementation due to the binding salts, which have resulted in an increase in modulus values. It can also be observed from Fig. 5 that keeping constant CED, the modulus values decay with an increase in strain level from 0.03 to 10%.

The above discussion explained the material behavior at large strain levels, which is absent at small strain levels (0.01% and lesser). The modulus values at the small strain levels tend to increase at different rates in all zones. The behavior at small strain levels is essentially linearly elastic in nature, while it is elasto-plastic at higher strain levels. The modulus value at small strain level increases with the increase in CED value. The increase in CED value is associated with the increase in density and cementation and/or decrease in the degree of saturation. A similar trend is also observed by Pintado X. et al.^49^, where the modulus values at low strain levels increase with the dry density and at lower degrees of saturation. The small strain levels apply to foundations subjected to dynamic loads and low-strain geophysical tests.

Using the mechanical behavior model plots, fundamental equations are developed comprising the relationship between CED and modulus values, E, at various strain levels where E is the secant modulus. The relationships for all the three zones of the plots for large and small strain levels are represented below:

2 $$ \begin{aligned} {\text{E}}_{{{\text{LI}}}} = & \left\{ {{1}.{2363}({1}.{7137}\varepsilon^{{ - 0.{184}}} ) \, \left( {{\text{CED}}} \right)^{{2}} {-}{ 1629}({1}.{6774}\varepsilon^{{ - 0.{163}}} ) \, \left( {{\text{CED}}} \right) \, + { 542692}({1}.{6838}\varepsilon^{{ - 0.{157}}} )} \right\} \\ \,\quad\quad / \, \Delta {\text{IDD }}/ \, \Delta {\text{p}}_{{\text{c}}} \\ \end{aligned} $$

3 $$ \begin{aligned} {\text{E}}_{{{\text{LII}}}} = & \left\{ {0.{2954}( - 0.{\text{762ln}}\left( \varepsilon \right) \, + \, 0.{4}0{73}) \, \left( {{\text{CED}}} \right)^{{2}} + { 575}({1}.{\text{8977ln}}\left( \varepsilon \right) \, - { 2}.{7568}) \, \left( {{\text{CED}}} \right) \, + { 567877}( - {2}.{\text{582ln}}\left( \varepsilon \right) \, + { 6}.{3925})} \right\} \\ \, / \, \Delta {\text{IDD }}/ \, \Delta {\text{p}}_{{\text{c}}} \\ \end{aligned} $$

4 $$ \begin{aligned} {\text{E}}_{{{\text{LIII}}}} = & \left\{ { - 0.0{5}0{2}( - 0.{\text{149ln}}\left( \varepsilon \right) \, + { 1}.{6255}) \, \left( {{\text{CED}}} \right)^{{2}} + { 754}( - 0.{\text{132ln}}\left( \varepsilon \right) \, + { 1}.{4661}) \, \left( {{\text{CED}}} \right) \, - { 1614985}( - 0.0{\text{78ln}}\left( \varepsilon \right) \, + { 1}.{3227})} \right\} \\ \, / \, \Delta {\text{IDD }}/ \, \Delta {\text{p}}_{{\text{c}}} \\ \end{aligned} $$

Equations (2) to (4) represent relationships for large strain levels. However, for small strain levels, Eqs. (5) to (7) can be used:

5 $$ \begin{aligned} {\text{E}}_{{{\text{SI}}}} = & \left\{ { - {2}.{9}0{73}( - {116}.{67}\varepsilon + {2}.{1667}) \, \left( {{\text{CED}}} \right)^{{2}} + { 9432}( - {81}.{835}\varepsilon + {1}.{8184}) \, \left( {{\text{CED}}} \right) \, - { 4314}0{26}( - {76}.{667}\varepsilon + {1}.{7667})} \right\} \\ & / \, \Delta {\text{IDD }}/ \, \Delta {\text{p}}_{{\text{c}}} \\ \end{aligned} $$

6 $$ \begin{aligned} {\text{E}}_{{{\text{SII}}}} = & \left\{ {0.{5496}( - {63}.{662}\varepsilon - 0.{3634}) \, \left( {{\text{CED}}} \right)^{{2}} + { 4324}({35}.{9}0{6}\varepsilon + 0.{64}0{9}) \, \left( {{\text{CED}}} \right) \, + { 1863674}( - {12}0.0{7}\varepsilon + 0.{2}00{7})} \right\} \\ & / \, \Delta {\text{IDD }}/ \, \Delta {\text{p}}_{{\text{c}}} \\ \end{aligned} $$

7 $$ \begin{aligned} {\text{E}}_{{{\text{SIII}}}} = & \left\{ {0.0{3}0{2}( - {47}.0{93}\varepsilon - 0.{5291}) \, \left( {{\text{CED}}} \right)^{{2}} + { 391}({23}.{987}\varepsilon + 0.{7613}) \, \left( {{\text{CED}}} \right) \, + {489681}0( - {14}.{845}\varepsilon + {1}.{1484})} \right\} \\ & / \, \Delta {\text{IDD }}/ \, \Delta {\text{p}}_{{\text{c}}} \\ \end{aligned} $$

The numbers in the subscripts in Eqs. (2) to (7) represent the respective zone of the plot whereas large and small strain levels are shown by L and S, respectively. CED represents the cohesive energy density in J/cm^3^, and ɛ is the strain level. As the simulated model has been constructed considering the natural composition of clay-bearing strata using a combination of clay and non-clay minerals with pore spaces, the strain, ɛ in Eqs. (2) to (7), represents the total strain of the clay-bearing strata. A linear interpolation could be performed for strain levels between 0.03% and 0.01%.

The CED can be calculated by Eqs. (8) to (12) obtained by Ahmed and Abduljauwad^7^ in their swelling behavior model as follows:

8 $$ {\text{CED }} = \, 0.0{625 }\left( {{\text{IWC}}} \right)^{{3}} {-}{ 3}.{575 }\left( {{\text{IWC}}} \right)^{{2}} + { 1}0.{5 }\left( {{\text{IWC}}} \right) + {283}0 + \Delta {\text{CED}}_{{{\text{CEC}}}} + \, \Delta {\text{CED}}_{{{\text{cat}}}} $$

where IWC is the initial water content. The correction due to CEC (ΔCED_CEC_) is then applied using the following expressions:

For CEC > 90 meq/100 g:

9 $$ \Delta {\text{CED}}_{{{\text{CEC}}}} = \, \left[ {0.0{717 }\left( {{\text{IWC}}} \right)^{{3}} {-}{ 3}.{775 }\left( {{\text{IWC}}} \right)^{{2}} {-}{ 22}.{917 }\left( {{\text{IWC}}} \right) + {3785}} \right]\left( {{\text{CEC}} - {9}0} \right)/\left( {{54}} \right) $$

For CEC < 90 meq/100 g:

10 $$ \Delta {\text{CED}}_{{{\text{CEC}}}} = \, - \left[ {0.000{2 }\left( {{\text{IWC}}} \right)^{{3}} + \, 0.{74 }\left( {{\text{IWC}}} \right)^{{2}} {-}{ 57}.{4171 }\left( {{\text{IWC}}} \right) + {1528}} \right]\left( {{9}0 - {\text{CEC}}} \right)/\left( {{36}} \right) $$

Equation (8) should also be corrected for binding/cementing salts and exchangeable cations (ΔCED_cat_) as follows:

11 $$ \begin{aligned} \Delta {\text{CED}}_{{{\text{cat}}}} = & \left[ {{71}00\left( {{\text{C}}/0.{1}} \right) + {725}0\left( {{\text{L}}/0.{1}} \right) + {5}0{5}0\left( {{\text{G}}/0.{2}} \right) + {3}0{1}0\left( {{\text{KCl}}/0.{1}} \right) + {325}0\left( {{\text{KP}}/0.{1}} \right) + {351}0\left( {{\text{D}}/0.{1}} \right) \, + {1}0{2}00\left( {{\text{P}}/0.{1}} \right)} \right] \\ & \left( {{1}0/{\text{IWC}}} \right)0.{85} + {\text{Ca}}\left( {{5}00} \right) + {\text{Mg}}\left( {{3}00} \right) + {\text{K}}\left( {{1}00} \right) \\ \end{aligned} $$

where C is Calcite; L is hydrated lime, G is Gypsum; KCl is potassium chloride fraction of Na-montmorillonite (smectite) content, and Ca is exchangeable calcium cation; Mg is exchangeable magnesium cation; P is Palygorskite; K is potassium exchangeable cation fraction of the total cations.

For CECs other than 54 meq/100 g, Eq. (8) is normalized as:

12 $$ {\text{CEDm }} = {\text{ e}}^{{(0.0{\text{1263x54}} + {\text{ln }}\left( {{\text{CED}}} \right) - 0.0{\text{1263CEC}})}} $$

Furthermore, a correction for the confinement effect, p_c,_ is also suggested in this study for the modulus values to account for the field stress conditions. This correction is given as:

13 $$ \Delta {\text{p}}_{{\text{c}}} = \, ({\text{p}}_{{\text{c}}} /{1}0{2})^{{0.{333}}} $$

The CEC of clay-bearing soils/rocks is controlled by the relative amount of swelling-clay minerals in the sample. Therefore, in addition to the correction for the confinement effect to get the final modified modulus value (E_m_), CEDm is also modified by the ratio of swelling-clay minerals to get the final CED value of the specimen for the mechanical behavior model.

### Up-scaling of the model

Based on the level of compaction or consolidation, natural or engineered clay-bearing strata contain varying proportion of micro to macropores. Percentages of macropores are almost minimal in the case of heavily overconsolidated/compacted clay-bearing soils/rocks owing to their high densities, while there could be relatively high percentages of macropores in low density strata. Therefore, the models formulated using molecular-level simulations may require an upscaling for the cases where macropores are predominantly present. In this study, therefore, a correction, ΔIDD in the form of upscaling has been applied to account for the macropores in the clay-bearing strata. The factor, ΔIDD represents the difference in densities of clay-bearing soils/rocks at the molecular and macroscopic levels due to the presence of macropores and is found to be ranging from about 1.0 to 3.0 depending upon the proportion of macropores in the strata. The upscaling mechanism is described below in the form of equations.

The initial dry density (IDD) of the clay-bearing strata resulting from molecular-level simulations is related to normalized CED value, CEDm, through the following correlations^7^:

14 $$ {\text{IDD}}_{{{\text{CEDm}}}} = \, \left( {0.00{\text{2CED}}_{{\text{m}}} {-}{ 1}.{3476}} \right){\text{ for CED}}_{{\text{m}}} < { 161}0 $$

15 $$ {\text{IDD}}_{{{\text{CEDm}}}} = \, \left( { - 0.0000{\text{8CED}}_{{\text{m}}} + { 2}.0{2}0{3}} \right){\text{ for CED}}_{{\text{m}}} > { 161}0{\text{ and }} < { 336}0 $$

16 $$  IDD_{{CEDm}}  = {\mkern 1mu} \left( { - 3{\text{ }} \times {\text{ }}10^{{ - 9}} \left( {CED_{m} } \right)^{2}  + {\mkern 1mu} 0.0002CED_{m}  + 1.1971} \right){\text{ }}for{\text{ }}CED_{m} {\text{ }} > 3360 $$

The factor, ΔIDD is defined as follows:

17 $$ \Delta {\text{IDD}} = {\text{IDD}}_{{{\text{CEDm}}}} /{\text{ IDD}}_{{{\text{specimen}}}} $$

where IDD_CEDm_ is the initial dry density from molecular-level simulations, and IDD_specimen_ is the initial dry density of strata. The modulus values obtained from the mechanical behavior model are reduced by the factor ΔIDD to account for macropores in the strata and to get final modulus values.

## Model validation

The model validation is mainly comprised of a testing program consisting of basic characterization tests, mineralogical study, and deformation properties, followed by the determination of CED and modulus values from the test results. Model validation has been carried out in two steps: (i) E-moduli validation at various strain levels from field and laboratory tests performed in this study and from an existing database, and (ii) validation through complete stress–strain plots.

### E-moduli validation from current study

The mechanical behavior model is validated through laboratory testing on natural swelling soil samples collected from clay-bearing strata and reconstituted laboratory specimens (control specimens) utilizing several proportions of soil constituents. Many soil constituents were selected to identify the utmost feasible composition of the natural clay-bearing strata. Thus, individual soil constituents were attained from various known sources to prepare control samples. Natural samples from clay-bearing strata were acquired from the Qatif and Hofuf areas of Saudi Arabia, known for their shallow subsurface heavily over consolidated clay/claystone deposits^50–53^. The large intact lumps and blocks of heavily over consolidated clay/claystone samples were obtained from the existing test pits and were wrapped with plastic sheets to maintain the natural moisture content of these samples. Laboratory compacted bentonite samples were prepared from the Bentonite collected from a known source in Saudi Arabia (Kanoo Est., KSA). Bentonite was taken out of bags, oven-dried for 24 h, and then put into air-tight containers for further sampling and testing. The control specimens were then prepared by static compaction in a compression machine.

Calcium carbonate was collected from Techno Pharmchem, India, and Gypsum samples were taken from Phosphate Plant, RIC, KSA. Sand samples were collected from the dunes in Jubail, KSA (Table 1) and mixed with Bentonite to formulate the control specimens with different CED.

**Table 1:** Gradation analysis of sand used in this study.

ASTM Sieve no	10	20	40	60	100	200
Size (mm)	2.0	0.85	0.425	0.25	0.15	0.075
Passing (%)	100	92	58	35	19	2

Basic characterization tests consisting of the grain size distribution (ASTM D6913), moisture content determination (ASTM D2216), and Atterberg limits (ASTM D4318) were performed on field-collected swelling clays and control specimens. Cation Exchange Capacity (CEC) tests were also carried out on swelling soils using Rayment & Higginson (1992) Method 15A2^54^. The results of basic characterization tests are summarized in Table 2.

**Table 2:** Summary of basic characterization tests.

Material	Designation	Source	Classification	Atterberg limits			CEC
				LL	PL	PI	
Bentonite	B	Kanoo Est., KSA	CH	442	85	357	71.3
Qatif Clay/Claystone	Q	Qatif, KSA	CH	157	54	103	51.0
Hofuf Clay/Claystone	H	Hofuf, KSA	CH	73	31	42	12.7
Calcium carbonate	C	Techno Pharmchem, India	–	–	–	–	–
Gypsum	G	Phosphate Plant, RIC, KSA	–	–	–	–	–
Sand	S	Jubail, KSA	SP	NP	NP	NP	–

CH, high-plasticity clay; SP, poorly graded sand; NP, non-plastic; LL, liquid limit; PL, plastic limit; PI, plasticity index.

X-ray diffraction (XRD) tests were performed using an Ultima IV X-Ray Diffractometer with copper radiation generated at 40 kV and 40 mA at the Research Institute (RI) in KFUPM. In this study, both quantitative and qualitative evaluations were performed to determine the type and amount of clay and non-clay minerals present in the samples. Randomly orientated samples (powdered specimens) were prepared by drying the sample in the oven and pressing it against the metal surface. Relative percentages of the different clay and non-clay minerals in natural clay-bearing strata of Hofuf and Qatif area and Bentonite are presented in Table 3.

**Table 3:** Relative amount of minerals in clay-bearing soil samples.

Clay type	Relative percentage (%)						
	Smectite	Palygorskite	Illite	Kaolinite	Calcite	Quartz	Gypsum
Bentonite	60	-	10	25	–	5	–
Qatif Clay/Claystone	32	7	21	–	22	9	9
Hofuf Clay/Claystone	3	5	35	–	40	17	–

**Figure 6:**
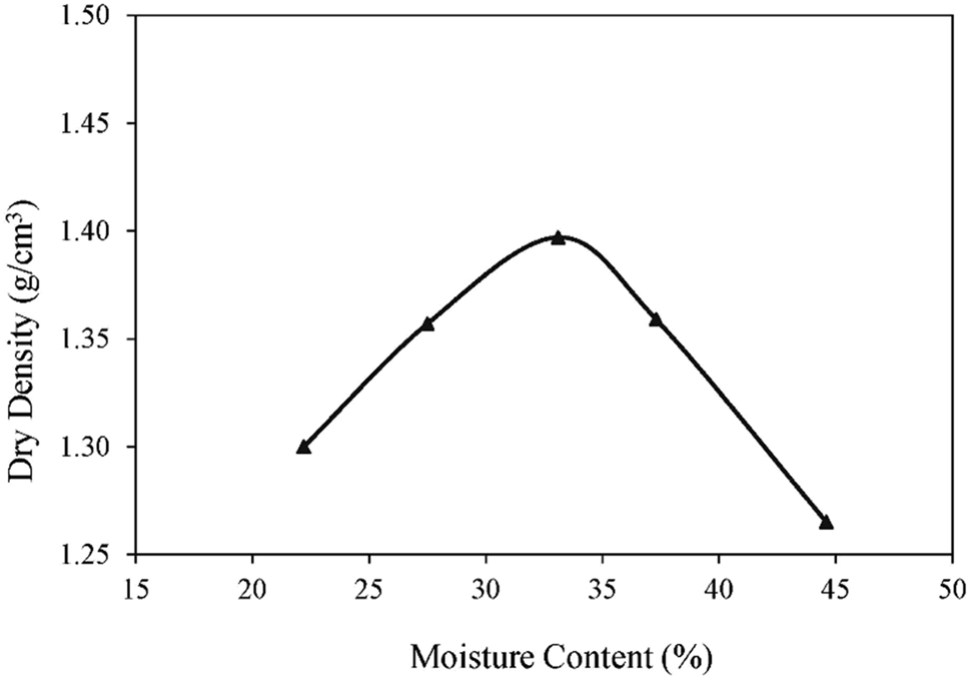
Moisture–density relationship (modified Proctor test).

**Figure 7:**
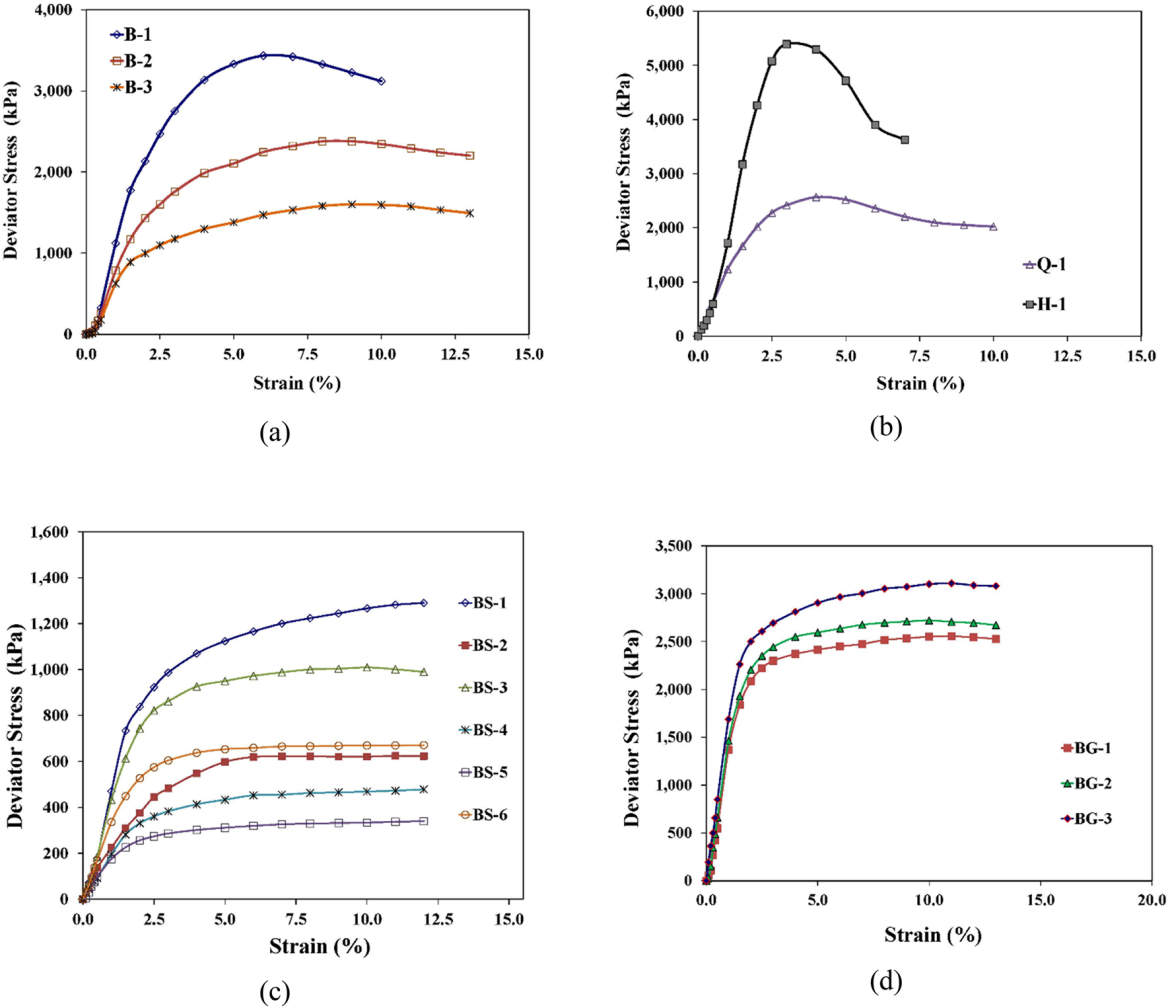
Stress–strain plots; (**a**) Bentonite samples on dry of optimum (B-1), optimum (B-2) and wet of optimum (B-3), (**b**) Swelling clay/claystone samples of Qatif and Hofuf at natural moisture content, (**c**) Bentonite mixed with sand, (**d**) Bentonite mixed with Gypsum.

To study the stress–strain response for the possible changes in fabric and structure, moisture-density relationships were established for the control specimens using the modified Proctor test procedures as per ASTM D1557. The results of the modified Proctor test are provided in Fig. 6. The control samples with recognized amounts of clay and non-clay minerals were arranged by static compaction in a compression machine at the moisture content and density on the "dry side of optimum," "optimum," and "wet side of optimum" using the results of the modified Proctor test to create different forms of fabric and structure. The undisturbed natural samples from clay-bearing strata were tested at the natural moisture contents and densities. The testing matrix for the experimental laboratory program carried out in this study is summarized in Table 4. These control and natural undisturbed specimens were then subjected to Unconsolidated Undrained (UU) Triaxial Compression tests as per ASTM D2850 to determine the stress-deformation properties. The stress–strain plots of UU Triaxial Compression tests for the controlled specimens and natural samples at a cell pressure of 300 kPa (the stress conditions that the material will face in the field under most project conditions) are shown in Fig. 7.

**Table 4:** Testing matrix for laboratory program carried out in this study.

Sample ID	Constituent type and percent	Moisture content state	Moisture content (%)	Initial dry density (g/cm^3^)
B-1	B100	Dry of OMC	25.00	1.327
B-2	B100	OMC	33.10	1.397
B-3	B100	Wet of OMC	39.90	1.327
BS-1	B90S10	Dry of OMC	25.00	1.327
BS-2	B70S30	Dry of OMC	25.00	1.327
BS-3	B90S10	OMC	33.10	1.397
BS-4	B80S20	OMC	33.10	1.397
BS-5	B70S30	OMC	33.10	1.397
BS-6	B90S10	Wet of OMC	39.90	1.327
BG-1	B90G10	OMC	33.10	1.397
BG-2	B85G15	OMC	33.10	1.397
BG-3	B80G20	OMC	33.10	1.397
BC-1	B85C15	Wet of OMC	39.90	1.327
Q-1	Clay/Claystone Samples	NMC	50.60	1.200
H-1	Clay/Claystone Samples	NMC	45.00	1.240

B, Bentonite; G, Gypsum; C, Calcite; S, sand; OMC, optimum moisture content; NMC, natural moisture content; Q, Qatif; H, Hofuf.

Crosshole seismic tests were performed as per ASTM D4428 using Olson CS Equipment on clay-bearing strata of the Hofuf area to validate the mechanical behavior model at low strain levels of 0.01% and lesser. In this test, three boreholes (spaced 3.0 m from center to center) were drilled in a straight line. The seismic waves were generated in one of the boreholes (called a "shot hole"), and its horizontal travel time was recorded in the other two boreholes (referred to as "receiver holes"). The holes were drilled and prepared for the tests before the testing by placing PVC pipes and grouting the annular space. The field procedure consisted of the generation of seismic waves in the shot hole and recording the time of arrival of waves in the receiver holes. The readings were recorded at 1.50 m, 2.0 m, 6.50 m, 7.0 m, and 7.50 m in swelling clay/claystone layers at two different locations. Based on the velocity of propagation of the waves (shear and compression) in the medium, the modulus values of the sub-surface layers were determined. A summary of the results of cross-hole tests is tabulated in Table 5.

**Table 5:** Crosshole seismic test results in clay-bearing strata of Hofuf.

Sample ID	Depth of Source & Receiver (m)	Distance from S to R1 (m)	Travel Time of wave from S to R1 (msec)		Distance from S to R2 (m)	Travel Time of wave from S to R2 (msec)		Average wave Velocity (m/sec)		Density **ρ** (g/cm^3^)	Poisson's ratio **υ**	Modulus **E** (M Pa)
			P	S		P	S	P	S			
H-2	1.50	3.00	1.61	5.07	6.00	3.62	11.78	1760	551	1.820	0.44	1595
H-3	2.00	3.00	1.62	5.09	6.00	3.59	11.91	1762	547	1.840	0.44	1591
H-4	6.50	3.00	1.58	5.38	6.00	3.47	11.62	1814	537	1.920	0.45	1608
H-5	7.00	3.00	1.49	5.22	6.00	3.61	11.44	1838	550	1.930	0.45	1692
H-6	7.50	3.00	1.49	5.17	6.00	3.56	11.39	1849	554	1.930	0.45	1716

S = Shot hole; R1 = First receiver hole; R2 = Second receiver hole; P = Primary wave; S = Secondary wave.

### E-moduli validation from existing literature

The molecular-level simulation results were validated from the existing literature using data from two (02) swelling clays (MX-80 and FEBEX Bentonite). The FEBEX and MX-80 are the bentonites used as Engineered Barrier systems in Finland, Sweden, and Spain. The mineralogical composition and material properties of both FEBEX (calcium bentonite) and MX-80 (sodium bentonite) samples have been presented in Tables 6 and 7. The information about MX-80 is taken from Kiviranta and Kumpulainen^55^ and Kiviranta et al.^56^, whereas the information about FEBEX properties is collected from Enresa^57^.

**Table 6:** Relative amount of minerals in FEBEX and MX-80.

Clay type	Relative percentage (%)					
	Smectite	Quartz	Plagioclase	Cristobalite	Calcite	K- feldspar
FEBEX	92 ± 3	2 ± 1	2 ± 1	2 ± 1	0.50	Traces
MX-80	88.2 ± 5.2	3.5 ± 0.1	2.9 ± 0.7	Traces	–	2.4 ± 1.50

**Table 7:** Material properties of FEBEX and MX-80.

Clay type	Cation exchange capacity (eq/kg)	Liquid limit (%)	Plasticity index (%)	Specific surface (m^2^/g)	Density of solid particles (g/cm^3^)
FEBEX	1.02	102	49	725	2.70
MX-80	0.86	500	450	624	2.78

Data on the mechanical properties of MX-80 and FEBEX bentonites were collected from Pintado X. et al.^49^. The resonant column tests on these bentonites were performed on different dry densities and degrees of saturation, and shear modulus G_max_ or G_0_ values were determined (Tables 8 and 9).

**Table 8:** Shear modulus values in FEBEX bentonite at p = 0.80 MPa.

Specimen ID	Initial moisture content (%)	Initial dry density (mg/m^3^)	Initial degree of saturation (%)	Go (GPa)
F-1	14.70	1.58	54	370
F-2	21.30	1.66	87	502
F-3	4.70	1.65	19	310
F-4	3.70	1.66	15	265
F-5	10.40	1.72	47	429
F-6	10.60	1.74	49	409
F-7	3.70	1.68	16	290
F-8	12.70	1.62	50	387

p, confinement pressure.

**Table 9:** Shear modulus values in MX-80 Bentonite at p = 0.80 MPa.

Specimen ID	Initial moisture content (%)	Initial dry density (mg/m^3^)	Initial degree of saturation (%)	Go (GPa)
MX-1	10.70	1.75	51	521
MX-2	19.00	1.56	69	409
MX-3	17.30	1.62	68	463
MX-4	24.70	1.58	92	253
MX-5	18.40	1.64	74	454
MX-6	21.10	1.68	89	303
MX-7	26.50	1.58	96	242
MX-8	17.00	1.61	67	394
MX-9	21.70	1.63	86	342
MX-10	15.40	1.66	63	449

p, confinement pressure.

### Stress–strain behavior model validation

The model developed in this study can be applied to predict stress–strain curves for clay-bearing soils/rocks. It can be used to predict the complete stress–strain behavior including elastic, elastoplastic, peak strength, and post peak behavior. Since the model is developed from the realistic simulations of the interaction between clay and non-clay minerals, pore water, and dissolved salts, it results in the stress–strain plots which constitute by default the elastic and elasto-plastic behaviors.

The following step-wise procedure is adopted to generate the stress–strain curves from the mechanical behavior model:

*Step 1* The nano-level model parameter, cohesive energy density (CED), at initial conditions of moisture content and dry density is determined using Eq. (8).

*Step 2* Eqs. (5) to (7) are used to find the modulus value for the initial sample conditions at low strain level of 0.001%.

*Step 3* The modulus values for successive strain increments of 0.01% to 10% are calculated using Eqs. (2) to (4).

*Step 4* During the sample shearing stage, the CED and density of the sample change in response to various strain levels. This change in CED and density for each strain increment can be determined by a factor equal to 10^–4^ × (E_2_-E_1_). The E-value for the next step is then determined against new CED and density values after each strain increment.

*Step 5* The deviator stress can then be obtained from E-values after each strain increment using Eq. (18), and results can be plotted as stress–strain curves.

18 $$E=\frac{{\upsigma }_{1}-{2\mathrm{\nu \sigma }}_{3}}{\upepsilon }$$

where σ_1_ and σ_3_ are the major and minor principal stresses, ν is Poisson's ratio, and ɛ is the strain.

## Results and discussions

### Hydro-mechanical behavior

Based on the mineralogical composition, CEC, initial moisture content, and density values, CED values were determined for the samples used in this study and also for MX-80 and FEBEX bentonites using the relationships presented in Eqs. (8) to (12) and the results are provided in Table 10. The modulus (E_i_) values for the natural undisturbed and control specimens of clay-bearing strata at any given strain level were estimated using stress–strain plots of UU triaxial compression tests (Fig. 7) using the hyperbolic function^58^.

**Table 10:** Cohesive energy density (CED) values.

Specimen details			Mineralogy							Exchangeable Cations					CED	
Sample ID	Initial water content (%)	Initial dry density (g/cm^3^)	Swelling clay minerals (%)	Palygorskite (%)	Non-clay / Non-swell (%)	Gypsum (%)	Fraction of swelling clay minerals	Calcite (%)	Fraction of swelling clay minerals	CEC	Na	Ca	Mg	K	CED (J/cm^3^)	CEDm (J/cm^3^)
B-1	25.00	1.327	70	0	30	0	0	0	0	71.3	0.76	0.14	0.10	0.10	1545	1330
B-2	33.10	1.397	70	0	30	0	0	0	0	71.3	0.76	0.14	0.10	0.10	1296	1130
B-3	39.90	1.327	70	0	30	0	0	0	0	71.3	0.76	0.14	0.10	0.10	1305	1138
BS-1	25.00	1.327	63	0	37	0	0	0	0	71.3	0.76	0.14	0.10	0.10	1545	1330
BS-3	33.10	1.397	63	0	37	0	0	0	0	71.3	0.76	0.14	0.10	0.10	1296	1130
BS-6	39.90	1.327	63	0	37	0	0	0	0	71.3	0.76	0.14	0.10	0.10	1305	1138
BG-1	33.10	1.397	63	0	37	10	0.06	0	0	71.3	0.76	0.14	0.10	0.10	1296	1783
BC-1	39.90	1.327	59.5	0	40.5	0	0	15	0.09	71.3	0.76	0.14	0.10	0.10	1305	3356
Q-1	50.60	1.200	53	7	40	9	0.048	22	0.132	51.0	0.50	0.50	0.00	0.00	1066	5278
H-1	45.00	1.240	38	5	57	0	0	40	0.17	12.7	0.50	0.50	0.00	0.00	611	9543
F-1	14.70	1.580	89	0	11	0	0	0.5	0.004	102	0.24	0.42	0.31	0.03	3046	2004
F-2	21.30	1.660	89	0	11	0	0	0.5	0.004	102	0.24	0.42	0.31	0.03	2542	1681
F-3	4.70	1.650	89	0	11	0	0	0.5	0.004	102	0.24	0.42	0.31	0.03	3607	2597
F-4	3.70	1.660	89	0	11	0	0	0.5	0.004	102	0.24	0.42	0.31	0.03	3635	2716
F-5	10.40	1.720	89	0	11	0	0	0.5	0.004	102	0.24	0.42	0.31	0.03	3338	2223
F-6	10.60	1.740	89	0	11	0	0	0.5	0.004	102	0.24	0.42	0.31	0.03	3326	2212
F-7	3.70	1.680	89	0	11	0	0	0.5	0.004	102	0.24	0.42	0.31	0.03	3635	2716
F-8	12.70	1.620	89	0	11	0	0	0.5	0.004	102	0.24	0.42	0.31	0.03	3189	2105
MX-1	10.70	1.750	83	0	17	0	0	0	0	86	0.61	0.27	0.10	0.02	2499	1779
MX-2	19.00	1.560	83	0	17	0	0	0	0	86	0.61	0.27	0.10	0.02	2089	1506
MX-3	17.30	1.620	83	0	17	0	0	0	0	86	0.61	0.27	0.10	0.02	2181	1567
MX-4	24.70	1.580	83	0	17	0	0	0	0	86	0.61	0.27	0.10	0.02	1787	1305
MX-5	18.40	1.640	83	0	17	0	0	0	0	86	0.61	0.27	0.10	0.02	2122	1528
MX-6	21.10	1.680	83	0	17	0	0	0	0	86	0.61	0.27	0.10	0.02	1975	1430
MX-7	26.50	1.580	83	0	17	0	0	0	0	86	0.61	0.27	0.10	0.02	1702	1248
MX-8	17.00	1.610	83	0	17	0	0	0	0	86	0.61	0.27	0.10	0.02	2197	1578
MX-9	21.70	1.630	83	0	17	0	0	0	0	86	0.61	0.27	0.10	0.02	1943	1408
MX-10	15.40	1.660	83	0	17	0	0	0	0	86	0.61	0.27	0.10	0.02	2281	1634

For MX-80 and FEBEX bentonites, the hardening soil model (Eq. 19) introduced in Plaxis^59^ was used to estimate the variation of G and the corresponding E with the strain level.

19 $$G\left(\gamma \right)= \frac{{G}_{o}}{1+0.385\frac{\gamma }{{\gamma }_{0.7}}}$$

In Eq. (19), γ_0.7_ corresponds to the strain at which the modulus G is 0.722G_o_. The following relationship, given by Pintado et al.^49^, was used for γ_0.7_ and shear modulus at a small strain.

20 $$ \gamma_{{0.{7}}} = { 2}.{6}0 \, \times { 1}0^{{ - {7}}} \times {\text{ G}}_{{\text{o}}}^{{2}} $$

**Figure 8:**
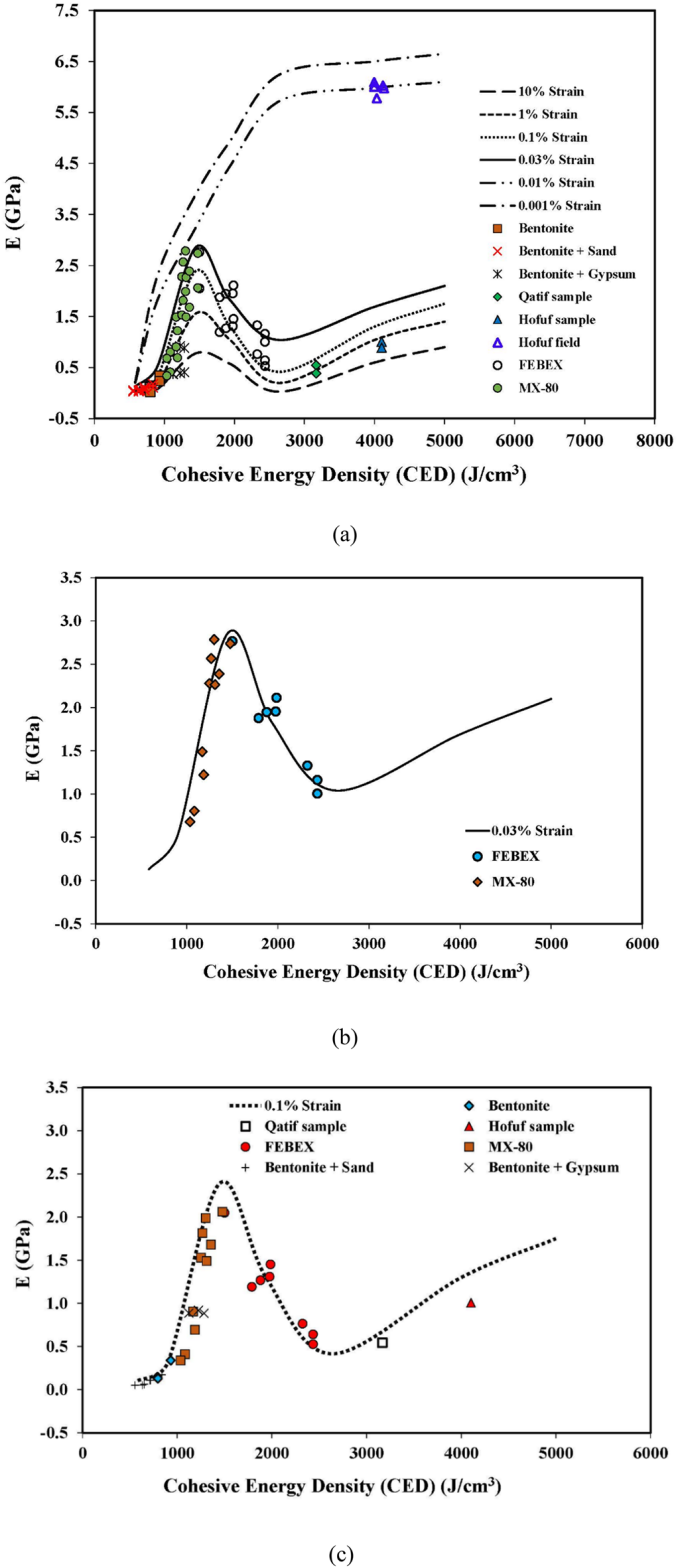

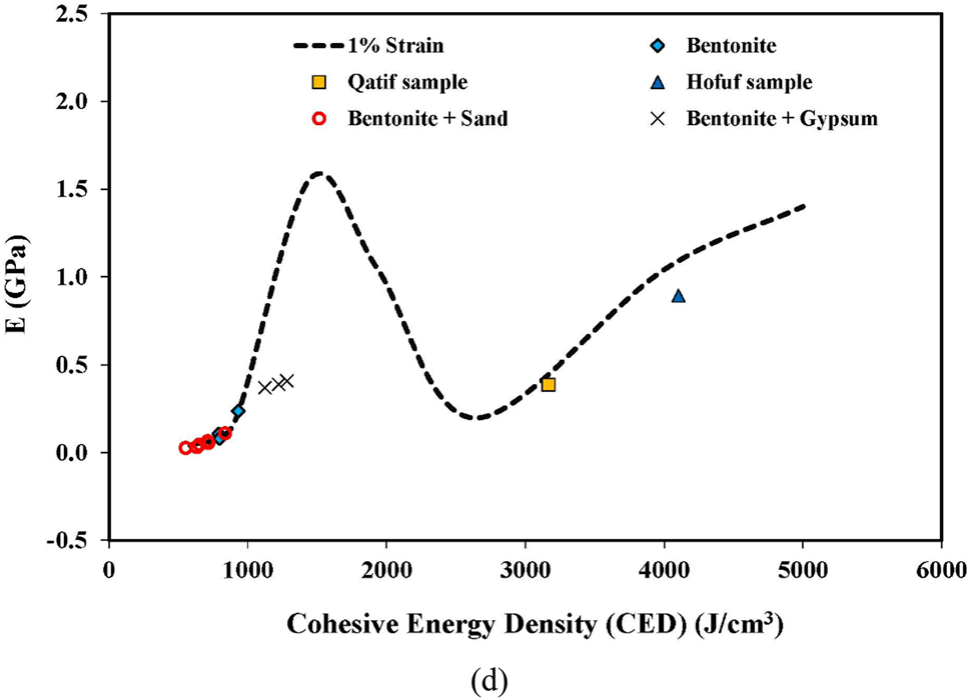
Mechanical behavior model validation; (**a**) Plot of all laboratory and field tests, (**b**) FEBEX and MX-80 samples on 0.03% strain level, (**c**) 0.10% strain level, (**d**) 1.0% strain level.

The modulus values were determined for all the tested specimens of natural and compacted clays, and the results are summarized in Table 11. Table 11 also includes the modulus values obtained from crosshole seismic tests, represented by sample IDs H-2 to H-6, to validate the mechanical behavior model at low strain levels. The estimated modulus values for two (02) swelling clays (MX-80 and FEBEX bentonite) against varying CED values are also presented in Table 12. The calculated modulus values were then plotted on the mechanical behavior model plots developed in this study (Fig. 8).

**Table 11:** Modulus determination from test results and comparison with mechanical model predictions.

Sample ID	Initial moisture content (%)	Initial dry density (g/cm^3^)	CEDm	Strain Level, ɣ (%)	E_i_ (kPa)	IDD_CEDm_ (g/cm^3^)	ΔIDD	Δp_c_	CEC Ratio	Final CED	E_m_ (GPa)	Model prediction, E_model_ (GPa)
B-1	25.00	1.327	1330	0.10	183,900	1.71	1.29	1.43	0.70	931	0.339	0.414
B-2	33.10	1.397	1130	0.10	117,300	1.23	0.88	1.43	0.70	791	0.148	0.191
B-3	39.90	1.327	1138	0.10	96,700	1.25	0.94	1.43	0.70	797	0.130	0.197
B-1	25.00	1.327	1330	1.00	127,800	1.71	1.29	1.43	0.70	931	0.236	0.206
B-2	33.10	1.397	1130	1.00	84,200	1.23	0.88	1.43	0.70	791	0.106	0.078
B-3	39.90	1.327	1138	1.00	59,300	1.25	0.94	1.43	0.70	797	0.080	0.082
B-3	39.90	1.327	1138	10.0	15,500	1.25	0.94	1.43	0.70	797	0.021	0.021
BS-1	25.00	1.327	1330	0.10	94,500	1.71	1.29	1.43	0.63	838	0.174	0.252
BS-2	25.00	1.327	1330	0.10	33,500	1.71	1.29	1.43	0.49	652	0.062	0.094
BS-3	33.10	1.397	1130	0.10	84,000	1.23	0.88	1.43	0.63	712	0.106	0.121
BS-6	39.90	1.327	1138	0.10	80,400	1.25	0.94	1.43	0.63	717	0.108	0.124
BS-1	25.00	1.327	1330	1.00	59,500	1.71	1.29	1.43	0.63	838	0.110	0.112
BS-4	33.10	1.397	1130	1.00	24,300	1.23	0.88	1.43	0.56	633	0.031	0.033
BS-6	39.90	1.327	1138	1.00	40,500	1.25	0.94	1.43	0.63	717	0.054	0.044
BG-1	33.10	1.397	1783	0.10	417,885	2.07	1.48	1.43	0.63	1123	0.888	0.928
BG-2	33.10	1.397	2055	0.10	437,250	2.05	1.47	1.43	0.60	1223	0.919	1.287
BG-1	33.10	1.397	1783	1.00	173,900	2.07	1.48	1.43	0.63	1123	0.370	0.517
BC-1	39.90	1.327	3356	0.10	151,400	1.93	1.46	1.43	0.60	1997	0.316	1.370
BC-1	39.90	1.327	3356	1.00	69,100	1.93	1.46	1.43	0.60	1997	0.144	0.945
Q-1	50.60	1.20	5278	0.10	182,800	2.50	2.08	1.43	0.60	3167	0.544	0.809
Q-1	50.60	1.20	5278	1.00	129,800	2.50	2.08	1.43	0.60	3167	0.387	0.546
H-1	45.00	1.24	9543	0.10	262,800	3.32	2.67	1.43	0.43	4103	1.007	1.386
H-1	45.00	1.24	9543	1.00	232,500	3.32	2.67	1.43	0.43	4103	0.891	1.026
H-2	44.70	1.26	9589	0.001	1,595,022	3.32	2.64	1.43	0.43	4123	6.028	7.113
H-3	44.50	1.27	9620	0.001	1,590,565	3.33	2.62	1.43	0.43	4137	5.974	7.119
H-4	46.10	1.31	9378	0.001	1,607,738	3.29	2.51	1.43	0.43	4033	5.778	7.072
H-5	46.70	1.32	9292	0.001	1,691,631	3.27	2.48	1.43	0.43	3995	6.005	7.055
H-6	46.70	1.32	9292	0.001	1,715,810	3.27	2.48	1.43	0.43	3995	6.091	7.055

E_i_, modulus value from field and laboratory tests; E_m_, final modified modulus value after corrections and upscaling.

**Table 12:** Modulus determination for FEBEX and MX-80 and comparison with mechanical model predictions.

Sample ID	Initial moisture content (%)	Initial dry density (g/cm^3^)	Degree of saturation (%)	CEDm	Go (MPa)	Strain Level, ɣ (%)	Strain Level, ɣ_0.7_ (%)	Gɣ (MPa)	E_i_ (MPa)	IDD_CEDm_ (g/cm^3^)	ΔIDD	Δp_c_	CEC Ratio	Final CED	E_m_ (GPa)	Model prediction, E_model_ (GPa)
F-1	14.70	1.58	54	2009	370	0.03	0.036	279	726	2.05	1.30	1.99	0.89	1788	1.875	2.003
F-2	21.30	1.66	87	1685	502	0.03	0.066	427	1110	2.08	1.25	1.99	0.89	1500	2.765	2.701
F-3	4.70	1.65	19	2612	310	0.03	0.025	212	551	2.00	1.21	1.99	0.89	2325	1.327	1.107
F-4	3.70	1.66	15	2735	265	0.03	0.018	162	422	1.99	1.20	1.99	0.89	2434	1.005	0.989
F-5	10.40	1.72	47	2231	429	0.03	0.048	346	899	2.03	1.18	1.99	0.89	1985	2.111	1.613
F-6	10.60	1.74	49	2220	409	0.03	0.043	323	840	2.03	1.17	1.99	0.89	1976	1.952	1.630
F-7	3.70	1.68	16	2735	290	0.03	0.022	190	493	1.99	1.18	1.99	0.89	2434	1.160	0.989
F-8	12.70	1.62	50	2111	387	0.03	0.039	298	776	2.04	1.26	1.99	0.89	1879	1.946	1.815
F-1	14.70	1.58	54	2009	370	0.10	0.036	178	462	2.05	1.30	1.99	0.89	1788	1.193	1.720
F-2	21.30	1.66	87	1685	502	0.10	0.066	316	822	2.08	1.25	1.99	0.89	1500	2.049	2.297
F-3	4.70	1.65	19	2612	310	0.10	0.025	122	317	2.00	1.21	1.99	0.89	2325	0.764	0.931
F-4	3.70	1.66	15	2735	265	0.10	0.018	85	222	1.99	1.20	1.99	0.89	2434	0.528	0.816
F-5	10.40	1.72	47	2231	429	0.10	0.048	238	618	2.03	1.18	1.99	0.89	1985	1.452	1.388
F-6	10.60	1.74	49	2220	409	0.10	0.043	217	564	2.03	1.17	1.99	0.89	1976	1.310	1.403
F-7	3.70	1.68	16	2735	290	0.10	0.022	105	273	1.99	1.18	1.99	0.89	2434	0.642	0.816
F-8	12.70	1.62	50	2111	387	0.10	0.039	195	506	2.04	1.26	1.99	0.89	1879	1.269	1.561
MX-1	10.70	1.75	51	1779	521	0.03	0.071	448	1164	2.07	1.19	1.99	0.83	1477	2.739	2.764
MX-2	19.00	1.56	69	1506	409	0.03	0.043	323	840	2.13	1.37	1.99	0.83	1250	2.279	1.847
MX-3	17.30	1.62	68	1567	463	0.03	0.056	384	997	2.28	1.41	1.99	0.83	1301	2.785	2.125
MX-4	24.70	1.58	92	1305	253	0.03	0.017	149	388	1.65	1.04	1.99	0.83	1083	0.804	1.080
MX-5	18.40	1.64	74	1528	454	0.03	0.054	374	971	2.18	1.33	1.99	0.83	1268	2.567	1.943
MX-6	21.10	1.68	89	1430	303	0.03	0.024	204	531	1.95	1.16	1.99	0.83	1187	1.223	1.530
MX-7	26.50	1.58	96	1248	242	0.03	0.015	138	358	1.51	0.96	1.99	0.83	1035	0.679	0.904
MX-8	17.00	1.61	67	1578	394	0.03	0.040	306	796	2.30	1.43	1.99	0.83	1310	2.263	2.176
MX-9	21.70	1.63	86	1408	342	0.03	0.030	248	644	1.90	1.16	1.99	0.83	1169	1.489	1.447
MX-10	15.40	1.66	63	1634	449	0.03	0.052	368	957	2.09	1.26	1.99	0.83	1356	2.388	2.451
MX-1	10.70	1.75	51	1779	521	0.10	0.071	337	876	2.07	1.19	1.99	0.83	1477	2.062	2.347
MX-2	19.00	1.56	69	1506	409	0.10	0.043	217	564	2.13	1.37	1.99	0.83	1250	1.530	1.398
MX-3	17.30	1.62	68	1567	463	0.10	0.056	274	712	2.28	1.41	1.99	0.83	1301	1.988	1.616
MX-5	18.40	1.64	74	1528	454	0.10	0.054	264	687	2.18	1.33	1.99	0.83	1268	1.816	1.473
MX-8	17.00	1.61	67	1578	394	0.10	0.040	202	524	2.30	1.43	1.99	0.83	1310	1.490	1.655
MX-9	21.70	1.63	86	1408	342	0.10	0.030	151	392	1.90	1.16	1.99	0.83	1169	0.907	1.085
MX-10	15.40	1.66	63	1634	449	0.10	0.052	259	673	2.09	1.26	1.99	0.83	1356	1.680	1.871

Based on these results, the CED values range from 1130 to 9543 J/cm^3^, with moisture contents varying from 25.0 to 50.6% for both natural and control samples. The test results presented in Table 10 dictate that the final CED values of the tested samples vary from 633 to 4103 J/cm^3^, and the modulus values range from 0.021 to 1.0 GPa at strain levels varying from 0.10% to 10.0%. The modulus values from low-strain crosshole tests were found to be 5.77 to 6.09 GPa against the final CED values of 3995 to 4137 J/cm^3^.

As discussed earlier, three zones could be identified from the mechanical behavior model plot, (i) Zone I between a CED value of 584 J/cm^3^ and 1450 J/cm^3^ in which the modulus values increase with an increase in CED, (ii) Zone II from a CED value of 1450 to 2640 J/cm^3^ where there is a tendency of reduction in the modulus values with the increase in CED, and (iii) Zone III having CED of more than 2640 J/cm^3^ and showing an increasing trend in the modulus values with the increase in CED due to cementation.

It can be observed from Fig. 8 that all the tested bentonite samples fall in Zone I. The final CED values of the tested bentonite samples range from 791 to 931 J/cm^3^. The modulus value at a 1.0% strain level and a CED of 791 J/cm^3^ is estimated as 0.106 GPa, whereas it is 0.236 GPa at a CED of 931 J/cm^3^. This confirms an increasing trend in the modulus values in Zone I with an increase in CED. The FEBEX bentonite samples are lying in Zone II of the mechanical model. The decrease in modulus values is observed in FEBEX bentonite samples with an increase in CED, which can be very well correlated with the lower moisture content of 4.70%, 3.70%, and 3.70% in samples F-3, F-4, and F-7, respectively. The undisturbed natural samples of swelling clays of Hofuf and Qatif areas fall in Zone III. The mineralogical composition of Hofuf and Qatif clays shows the presence of Gypsum and Calcite (Table 6) in the samples. The CED values of Hofuf and Qatif clays are calculated as 4103 J/cm^3^ and 3167 J/cm^3^, respectively. The higher CED values in Zone III are caused by the increase in cementation due to binding salts, which increases the stiffness values with CED. The modulus values of Hofuf and Qatif clays are estimated to be 0.891 GPa and 0.387 GPa, respectively, at a 1.0% strain level, validating an increasing trend of the modulus values with CED in Zone III.

A comparison is also provided in Tables 11 and 12 between the laboratory and field-determined modulus values from crosshole tests and the ones predicted by the mechanical behavior model through Eqs. (2) to (7). It can be seen from the results that the modulus values of the tested samples at strain levels of 0.001 to 10% are in very close agreement with the model predictions. However, a major discrepancy was seen for the bentonite samples with Calcite as a cementing agent, which can be ascribed to the relatively low solubility of Calcite in distilled water.

Typically, foundations of civil engineering structures are placed in partially saturated soil layers. The moisture levels of the subsurface strata often increase after construction due to the reduced evaporation, percolation of surface water(s), and the potential of leakage from utilities. An increase in moisture level in the clay-bearing strata below foundations results in the swelling strains and associated reduction in the CED value depending upon the exchangeable cations, CECs, and binding/cementing agents present in the strata. In response to the swelling strains, the modulus value also decreases as a function of the difference between initial and final densities with respect to changes in CED value. This reduction in modulus value causes additional compressive strains under the constant superimposed loads from the structure. The net result of these coupled swelling and compressive strains defines the overall hydro-mechanical response of clay-bearing strata under the foundations. The overall hydro-mechanical response is formulated through the coupling of the mechanical behavior model developed in this study with the existing nano-level swell behavior model by Ahmed and Abduljauwad^7^. The swell potential can be determined using the swell behavior model^7^ from the following Equation:

21 $$ {\text{Swell }}\left( \% \right) \, = \, \left( {{\text{FDD}} - {\text{IDD}}} \right)/{\text{FDD }} \times { 1}00 $$

The initial dry densities (IDD) are calculated using Eq. (13) to Eq. (15). The final dry densities (FDD) are related to CEDm through the following correlations^7^:

22 $$ {\text{FDD}}_{{{\text{CEDm}}}} = \, \left\{ {{\text{IDD }} + \, \left[ {\left( {0.00000{\text{7CED}}_{{\text{m}}} + \, 0.{3443}} \right) \, {-}{\text{ IDD}}} \right]} \right\} \, \times \, \left( {{1}0{2}/{\text{P}}} \right)^{{0.{5}}} {\text{CED}}_{{\text{m}}} {\text{for CED}}_{{\text{m}}} < { 161}0 $$

23 $$ {\text{FDD}}_{{{\text{CEDm}}}} = \, \left\{ {{\text{IDD }} + \, \left[ {\left( {0.000{\text{5CED}}_{{\text{m}}} - \, 0.{4517}} \right) \, {-}{\text{ IDD}}} \right]} \right\} \, \times \, \left( {{1}0{2}/{\text{P}}} \right)^{{0.{5}}} {\text{CED}}_{{\text{m}}} {\text{for CED}}_{{\text{m}}} > { 161}0{\text{ and }} < { 336}0 $$

24 $$ {\text{FDD}}_{{{\text{CEDm}}}} = \, \left\{ {{\text{IDD }} + \, \left[ {\left( {0.0000{\text{6CED}}_{{\text{m}}} + { 1}.00{82}} \right) \, {-}{\text{ IDD}}} \right]} \right\} \, \times \, \left( {{1}0{2}/{\text{P}}} \right)^{{0.{5}}} {\text{CED}}_{{\text{m}}} {\text{for CED}}_{{\text{m}}} > { 336}0 $$

where FDD is in units of g/cm^3^, CED is in J/cm^3^, and P, seating/confining pressure, is in kPa.

Incremental swelling can be estimated on each incremental intake of water content using the slope = (IDD-FDD)/(FWC-IWC). Final water content can also be assessed from the swelling behavior model as:

25 $$ {\text{FWC }} = \, 0.0{\text{789CED}}_{{{\text{vw}}}} + { 56}.{\text{648 for CED}}_{{{\text{vw}}}} < \, - {87} $$

26 $$ {\text{FWC }} = \, 0.00{39}\left( {{\text{CED}}_{{{\text{vw}}}} } \right)^{{2}} + { 1}.0{\text{587 CED}}_{{{\text{vw}}}} + { 1}0{6}.{\text{44 for CED}}_{{{\text{vw}}}} > \, - {87} $$

where FWC, final water content, is in the units of percent and CED_vw_ is in J/cm^3^ and can be calculated from Eq. (27) given by Ahmed and Abduljauwad^7^.

27 $$ {\text{CED}}_{{{\text{vw}}}} =_{{}} - 0.0{\text{81IWC}}^{{2}} + {7}.{\text{5421IWC}} - {131}.{1 } + \, \left( { - {\text{162G}}/0.{2}} \right) \, + \, \left( { - {\text{362C}}/0.{1}} \right) $$

**Figure 9:**
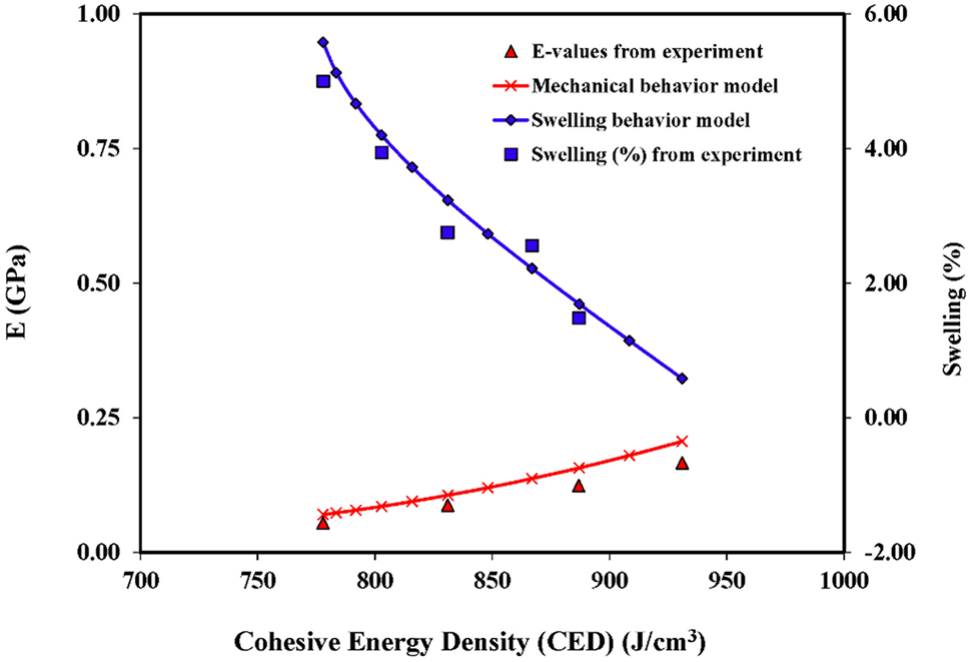
Coupled hydro-mechanical behavior and its validation.

An illustration of the coupled hydro-mechanical behavior is made using a general case of a shallow foundation placed in a partially-saturated, clay-bearing strata (Bentonite in this case) with initial conditions of IWC = 25% and IDD = 1.327 g/cm^3^. The saturation level in the clay-bearing strata is considered to increase from an initial water content of 25% to a final water content value of 35% after construction. The swell potential calculated through the swelling behavior model in ten equal increments of moisture content is presented in Table 13. A typical characteristic strain of 1.0% is considered for the modulus determination through Eq. (2) using the mechanical behavior model, and values are provided in Table 13 corresponding to each increment in water content. The results are also presented in Fig. 9, showing a decrease in CED values upon swelling and the corresponding reduction in the modulus values. The reduction in the modulus values from 0.206 GPa to 0.073 GPa against 5.13% swelling will cause additional compressive strains under the constant load from the structure. The foundation will finally be subjected to the net result of these coupled swelling and compressive strains.

**Table 13:** Coupled hydro-mechanical behaviour.

Sample parameters				CED			Swell behavior model				Mechanical behavior model		
Initial Water Content (%)	Initial Dry Density (g/cm^3^)	Incremental Final Water Content (%)	Confining Pressure, P (kPa)	CED (J/cm^3^)	CEDm (J/cm^3^)	CEDvw (J/cm^3^)	Terminal Final Water content (%)	Terminal Final Dry Density (g/cm^3^)	Incremental Final Dry Density (g/cm^3^)	Swell (%) at Confining Pressure, P	Final CED	Strain Level (%)	E_model_ (GPa)
25	1.327	26	102	1545	1330	7	114	0.354	1.316	0.58	931	1.0	0.206
26	1.316	27	102	1505	1298	10	118	0.353	1.306	1.15	909	1.0	0.180
27	1.306	28	102	1467	1267	13	121	0.353	1.295	1.69	887	1.0	0.157
28	1.295	29	102	1431	1238	17	125	0.353	1.286	2.22	867	1.0	0.137
29	1.286	30	102	1398	1212	19	129	0.353	1.276	2.73	848	1.0	0.120
30	1.276	31	102	1367	1187	22	132	0.353	1.267	3.23	831	1.0	0.106
31	1.267	32	102	1340	1166	25	135	0.352	1.259	3.72	816	1.0	0.095
32	1.259	33	102	1317	1147	27	138	0.352	1.250	4.20	803	1.0	0.086
33	1.250	34	102	1298	1131	30	141	0.352	1.242	4.67	792	1.0	0.079
34	1.242	35	102	1283	1119	32	144	0.352	1.234	5.13	784	1.0	0.073

The validation of coupled behavior through experimental data is also presented in Fig. 9. The UU Triaxial Compression and swelling-potential tests (ASTM D4546) were performed on the samples prepared at an initial moisture content of 25% and then allowed to swell by adding water in increments of 1% to reach a final moisture content of 35%. The predictions of swelling percent and modulus values are in well agreement with the values obtained from test results.

The swelling behavior model proposed by Ahmed and Abduljauwad^7^ has been verified using several laboratory control samples and the undisturbed samples acquired from the field in various studies carried out by Ahmed and Abduljauwad^7,60,61^. This model has also been successfully used by Abduljauwad and Ahmed^62^ for the prediction of swelling of field clay deposits.

### Stress–strain behavior prediction

**Figure 10:**
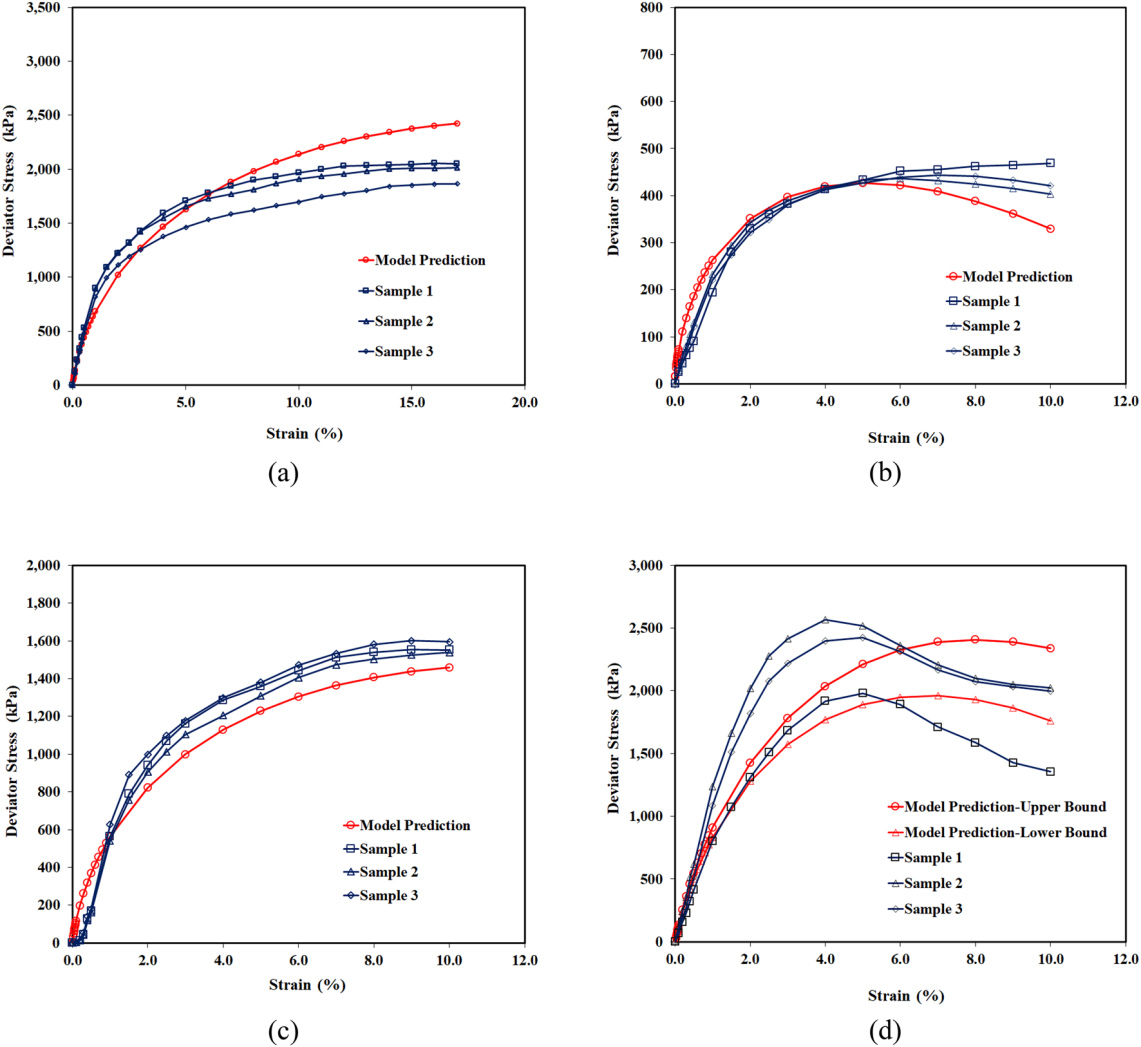
Model predictions of stress–strain plots; (**a**) Bentonite samples on dry side of optimum, (**b**) Bentonite samples mixed with 20% sand at optimum moisture content, (**c**) Bentonite samples on wet side of optimum, (**d**) Clay/claystone samples of Qatif at natural moisture content.

The model has been successfully applied to predict stress–strain curves for swelling soils. Figure 10 shows the stress–strain curves predicted from the model compared with the experimental curves generated from UU Triaxial Compression Tests on both natural and controlled samples. The stress–strain curves predicted from the mechanical behavior model captured successfully the main features of the stress–strain response of clay-bearing strata, including the initial elastic response, plasticity, peak strength, and post peak behavior. The experimental curves were obtained for three (03) replicated specimens of each set of conditions to observe the variation in the stress–strain behavior among the tested specimens. The tests on reconstituted replicated specimens have resulted in similar stress–strain plots due to the controlled sample parameters. The natural clay/claystone samples, however, have shown little variation in the peak stress and the corresponding strain values. This variation in peak stress is associated with the natural variation of several minerals quantified in the field samples of the Qatif clay/claystone. The variation in mineralogy is presented by upper and lower bound model predictions against the maximum and minimum gypsum content of 12% and 9% in the field samples, respectively.

## Conclusions

In molecular-level simulations, a matrix of clay-bearing strata constituting both clay and non-clay particles, pore water, and dissolved salts was created to model interactions among these particles. The results of molecular-level simulations were compiled to develop a hydro-mechanical model that can be utilized to predict the swelling-mechanical behavior of the clay-bearing strata. The model can be applied to all possible combinations of clay and non-clay minerals with cementing agents.

The study further concludes that the coupled hydro-mechanical response under given loading conditions can be well assessed through the coupling of the mechanical behavior model developed in this study with the existing nano-level swell behavior model by Ahmed and Abduljauwad^7^.

The proposed model can be effectively used to predict the overall material behavior of clay-bearing strata, covering all possible variations in the structure and fabric of clay-bearing soils/rocks having nano, micro and macropores. The model is characterized by a set of few parameters only, including cation exchange capacity, density, moisture content, and cohesive energy density, along with field stress conditions. These parameters can be determined from the basic characterization tests on the representative samples with great accuracy.

The predictions of stiffness moduli and stress–strain plots from the proposed model have been found to be in well agreement with the results obtained from the macro-level tests on the field and laboratory samples. Since the model is developed from the realistic simulations of the interaction between clay and non-clay minerals, pore water, and dissolved salts, it results in the stress–strain plots which constitute the elastic and elasto-plastic behaviors. The developed model has also been successfully validated from the existing literature using the data from two (02) swelling clays (MX-80 and FEBEX Bentonite). This suggests that the model can be used in all fields, such as civil engineering, agriculture, the petroleum industry, and waste management.

## Data Availability

Data generated or analyzed during this study are provided in full within the published article.
